# GCN5 contributes to intracellular lipid accumulation in human primary cardiac stromal cells from patients affected by Arrhythmogenic cardiomyopathy

**DOI:** 10.1111/jcmm.17396

**Published:** 2022-06-16

**Authors:** Chiara Volani, Alessandra Pagliaro, Johannes Rainer, Giuseppe Paglia, Benedetta Porro, Ilaria Stadiotti, Luisa Foco, Elisa Cogliati, Adolfo Paolin, Costanza Lagrasta, Caterina Frati, Emilia Corradini, Angela Falco, Theresa Matzinger, Anne Picard, Benedetta Ermon, Silvano Piazza, Marzia De Bortoli, Claudio Tondo, Réginald Philippe, Andrea Medici, Alexandros A. Lavdas, Michael J.F. Blumer, Giulio Pompilio, Elena Sommariva, Peter P. Pramstaller, Jakob Troppmair, Viviana Meraviglia, Alessandra Rossini

**Affiliations:** ^1^ Institute for Biomedicine Eurac Research Affiliated Institute of the University of Lübeck Bolzano Italy; ^2^ 9304 The Cell Physiology MiLab Department of Biosciences Università degli Studi di Milano Milano Italy; ^3^ 9305 School of Medicine and Surgery Università degli Studi di Milano‐Bicocca Vedano al Lambro MB Italy; ^4^ Unit of Vascular Biology and Regenerative Medicine Centro Cardiologico Monzino IRCCS Milano Italy; ^5^ Fondazione Banca dei Tessuti di Treviso Treviso Italy; ^6^ Department of Medicine and Surgery Università degli Studi di Parma Parma Italy; ^7^ Department of Cellular, Computational and Integrative Biology – CIBIO Università degli Studi di Trento Povo TN Italy; ^8^ Computational Biology International Centre for Genetic Engineering and Biotechnology ICGEB Trieste Italy; ^9^ Heart Rhythm Center Centro Cardiologico Monzino IRCCS Milano Italy; ^10^ 9304 Department of Biomedical Surgical and Dental Sciences Università degli Studi di Milano Milano Italy; ^11^ 9304 Department of Clinical Electrophysiology&Cardiac Pacing Università degli Studi di Milano Milano Italy; ^12^ Daniel Swarovski Research Laboratory Department of Visceral, Transplant and Thoracic Surgery Medical University Innsbruck Innsbruck Austria; ^13^ Department of Anatomy, Histology and Embryology Institute of Clinical and Functional Anatomy Medical University Innsbruck Innsbruck Austria

**Keywords:** Arrhythmogenic cardiomyopathy, cellular redox mechanisms, histone acetyltransferase GCN5, human cardiac stromal cells, intracellular lipid accumulation, reactive oxygen species

## Abstract

Arrhythmogenic cardiomyopathy (ACM) is a genetic disease associated with sudden cardiac death and cardiac fibro‐fatty replacement. Over the last years, several works have demonstrated that different epigenetic enzymes can affect not only gene expression changes in cardiac diseases but also cellular metabolism. Specifically, the histone acetyltransferase GCN5 is known to facilitate adipogenesis and modulate cardiac metabolism in heart failure. Our group previously demonstrated that human primary cardiac stromal cells (CStCs) contribute to adipogenesis in the ACM pathology. Thus, this study aims to evaluate the role of GCN5 in ACM intracellular lipid accumulation. To do so, CStCs were obtained from right ventricle biopsies of ACM patients and from samples of healthy cadaveric donors (CTR). GCN5 expression was increased both in *ex vivo* and *in vitro* ACM samples compared to CTR. When GCN5 expression was silenced or pharmacologically inhibited by the administration of MB‐3, we observed a reduction in lipid accumulation and a mitigation of reactive oxygen species (ROS) production in ACM CStCs. In agreement, transcriptome analysis revealed that the presence of MB‐3 modified the expression of pathways related to cellular redox balance. Altogether, our findings suggest that GCN5 inhibition reduces fat accumulation in ACM CStCs, partially by modulating intracellular redox balance pathways.

## 1 INTRODUCTION

The normal human adult heart is composed by cardiomyocytes and non‐cardiomyocyte cells. The latter represent the major part in terms of total cell number, and, among them, cardiac mesenchymal stromal cells (CStCs, including cardiac fibroblasts) are the vast majority.^1^ CStCs play a crucial role in multiple aspects of myocardial function, including synthesis and deposition of extracellular matrix (ECM), and cell–cell communication.^2^ However, during pathological changes, CStCs impede optimal electrical conduction and prompt to deposition of fibrotic tissue.^3^

Arrhythmogenic cardiomyopathy (ACM, OMIM: 609040) is a genetic cardiac disease characterized by structural and functional alterations, mainly in the right ventricle.^4^ The progressive loss of cardiomyocytes, associated with fibro‐fatty replacement of the myocardium,^5^ results in the alteration of the normal electrical impulse conduction and in a consequent increased risk of ventricular arrhythmias and sudden cardiac death (SCD).^6^ For years, ACM has been considered a cardiomyocyte disease; however, we have recently demonstrated that CStCs contribute to the disease pathophysiology.^7^ In fact, CStCs from ACM patients accumulate more lipid droplets than CStCs isolated from healthy control specimens, when exposed to a trigger medium (adipogenic medium).^7^

Of note, a key issue in ACM pathogenesis remains the fibro‐fatty myocardial replacement that can contribute to different disease prognosis.^8^ To date, ACM treatments involve the management and prevention of symptoms, based on anti‐arrhythmic drugs, implantable cardioverter defibrillator (ICD) insertion and, for worst cases, heart transplantation.^9^ There is no available therapy that directly targets the fibro‐fatty substitution.

Over the last ten years, several works have demonstrated that different epigenetics enzymes such as histone acetyltransferases (HATs) play an important role in governing gene expression changes underlying cardiac diseases.^10^ Importantly, it has been reported that HATs can modulate the function of many proteins involved in the regulation of cellular signalling and energy metabolism, including enzymes of glycolysis, glucose oxidation, electron transport chain and fatty acid β‐oxidation.^11^ General control non‐repressed 5 protein (GCN5), encoded by the *KAT2A*/*GCN5* gene, is a histone acetyltransferase belonging to the GNAT superfamily^12^, ^13^ that plays a role in cell proliferation, differentiation and DNA repair.^14^, ^15^ GCN5 also regulates cell energetic state and lipid metabolism.^16^

In this paper, we evaluated the possible involvement of GCN5 in the intracellular lipid accumulation observed in ACM CStCs. Our data indicate that GCN5 inhibition results in a reduced fat accumulation, modulated in part by the action on intracellular redox processes.

## 2 METHODS

### 2.1 Ethics statement

The present study was conducted in accordance with the Declaration of Helsinki and approved by the ethical committee of the Centro Cardiologico Monzino IRCCS (07/06/2012) and the South Tyrol Azienda Sanitaria (13/03/2014, N.1/2014). Written informed consent was obtained from all participants.

### 2.2 Study patient characteristics

Right ventricle samples were obtained from 16 patients affected by ACM undergoing catheter biopsies for diagnostic purposes, as previously described.^17^, ^18^ Table 1 summarizes the main clinical and genetical characteristics of the patients enrolled in this study. Ventricular samples from individuals not affected by ACM (CTR) were obtained from 7 cadaveric donors (accidental death), provided by ‘Fondazione Banca dei Tessuti di Treviso’.

**TABLE 1:** Clinical characteristics of ACM patients. ACM patients were categorized by adherence to major or minor diagnostic criteria according to the 2010 International Task Force.^64^ VT: ventricular tachycardia; PVC: premature ventricular contraction; ab: abnormalities. Only pathogenic or likely pathogenic variants found in the genes *DSC2*, *DSG2*, *DSP*, *PKP2*, *JUP*, *TMEM43*, *DES*, *RYR2*, *PLN*, *SCN5A* and *LMNA* are reported in the table

ACM patient ID	Onset: type/age	Dysfunction and structural alterations	Tissue characterization of wall	Repolarization ab.	Depolarization/conduction ab.	Arrhythmias	Family history	Genetics
ACM 1	VT/42	Major	Not conclusive	Major	Negative	Major	Major	*PKP2* c.1643delG p.G548Vfs*15
ACM 2	PVC/27	Major	Not conclusive	Major	Negative	Minor	Major	*PKP2* c.2013delC p.K672Rfs*12
ACM 3	VT/50	Minor	Major	Negative	Minor	Major	Negative	Negative
ACM 4	PVC/56	Minor	Minor	Negative	Minor	Minor	Negative	Negative
ACM 5	VT/35	Minor	Major	Negative	Minor	Major	Major	*PKP2* c.2013delC p.K672Rfs*12
ACM 6	VT/63	Major	Not conclusive	Major	Minor	Minor	Negative	negative
ACM 7	PVC/34	Major	Minor	Negative	Minor	Minor	Negative	*PKP2* c.548G>A p.S183N
ACM 8	PVC/47	Minor	Not conclusive	Major	Major	Major	Negative	*DSP* c.88G>A p.V30M
ACM 9	VT/24	Major	Not conclusive	Minor	Negative	Major	Negative	*DSG2* c.1003A>G p.T335A
ACM 10	ECG alterations/41	Major	Not conclusive	Major	Negative	Minor	Negative	n.a.
ACM 11	VT/28	Minor	Minor	Minor	Negative	Major	Negative	n.a.
ACM 12	syncope/54	Minor	Minor	Major	Negative	Major	Negative	n.a.
ACM 13	VT/44	Minor	Not conclusive	Major	Negative	Minor	Negative	n.a.
ACM 14	VT/51	Major	Major	Minor	Negative	Major	Major	*PKP2* c.1643delG p.G548Vfs*15
ACM 15	ECG alterations/26	Major	Major	Major	Negative	Minor	Negative	*PKP2* c.2569_3018del50 R857fs*1
ACM 16	VT/34	Major	Major	Major	Negative	Major	Negative	Negative

### 2.3 Genetics of ACM‐associated genes

Variants in *DSC2*, *DSG2*, *DSP*, *PKP2*, *JUP*, *TMEM43*, *DES*, *RYR2*, *PLN*, *SCN5A* and *LMNA* were analysed as previously described^8^ and the pathogenicity classified according to Richards et al.^19^

### 2.4 Cardiac stromal cells culture and adipogenic differentiation

Cardiac stromal cells (CStCs) from ACM patients and CTR individuals were obtained as previously reported.^7^, ^20^ Cells were cultured in basal medium composed of IMDM (Lonza), supplemented with 20% FBS (Hyclone, Italy), penicillin‐streptomycin 1% (Thermo Fisher Scientific) and 20 mM L‐Glutamine (Thermo Fisher Scientific). Adipogenic differentiation was performed by culturing the cells for 7 days in adipogenic medium (ADIPO) composed of IMDM, supplemented with 10% FBS (Gibco), 0.5 mM IBMX (Sigma‐Aldrich), 0.1 mM indomethacin (Sigma–Aldrich), 0.1 µM hydrocortisone (Sigma–Aldrich), penicillin‐streptomycin 1% (Thermo Fisher Scientific) and 20 mM L‐Glutamine (Thermo Fisher Scientific).^7^ CStC treatment with the specific GCN5 inhibitor MB‐3 (200 µM, Sigma‐Aldrich)^21^, ^22^ was performed in ADIPO medium for 7 days.

### 2.5 Lentiviral transduction for GCN5 knockdown

Lentiviral particles expressing hairpin RNA (shRNA) targeting *GCN5* and Green Fluorescent Protein (GFP) (ORIGENE) were used to induce GCN5 knockdown in ACM CStCs. 7500 cells/cm^2^ were plated and cultured for 72 h in basal medium. ACM CStCs were transduced with 100 MOI (Multiplicity of Infection) of lentiviral particles in ADIPO supplemented with 10 µg/ml polybrene (Merck). Transduction medium was refreshed after 72 h, and cells were cultured in ADIPO for additional 4 days. A scramble shRNA with a C‐terminal monomeric GFP was used as a control. After 7 days, cells were collected for protein analysis to confirm GCN5 knockdown and fixed to evaluate the effect of GCN5 knockdown on lipid accumulation.

### 2.6 Western Blot analysis

Cells were washed in phosphate‐buffered saline (PBS, Thermo Fisher Scientific) and lysed using protein extraction buffer (10 mM Tris‐HCl pH 7.4, 150 mM NaCl, 1% sodium deoxycholate (NaDoc), 0.1% sodium dodecyl sulphate (SDS) and 1% glycerol) supplemented with protease and phosphatase inhibitor mix (Roche). Total lysates were quantified using BCA Protein Assay Kit (Life Technologies). 15 µg of proteins was separated by SDS‐PAGE on precast gradient (4–12%) gels (Invitrogen) and transferred onto nitrocellulose membrane in Transfer buffer (Invitrogen) supplemented with 10% (vol/vol) methanol. Membranes were blocked in PBS 0.05% Tween 5% non‐fat dry milk for 1 h at room temperature (RT) and then incubated overnight at 4°C with anti‐GCN5 (1:1000, Cell Signaling, #3305, rabbit) and anti‐GAPDH (1:5000, Santa Cruz, Sc‐32233, mouse). After washing, membranes were incubated with the appropriate HRP‐conjugated secondary antibody for 1 h at RT. Detection was performed using the enhanced chemiluminescence system (ECL, Pierce™ ECL Western Blotting Substrate kit). Images were acquired with the ChemiDoc MP Imaging System (Bio‐rad) and quantified using Image Lab software 5.2.1 (Bio‐Rad).

### 2.7 Intracellular Lipid Staining analysis

After 7 days of adipogenic differentiation, cells were fixed with 4% (vol/vol) paraformaldehyde (PFA) for 15 min at RT and then incubated with 0.5 µM BODIPY^®^ 493/503 (Thermo Fisher Scientific) diluted in PBS or HCS LipidTOX™ Deep Red Neutral Lipid Stain (Thermo Fisher Scientific) 1X diluted in PBS for 20 min at room temperature (RT) in the dark. HCS LipidTOX™ Deep Red Neutral Lipid Stain was used only for GCN5 knockdown experiment in ACM CStCs transduced with GFP‐tagged lentiviral particles, in order to avoid spectral overlap between GFP and BODIPY^®^ 493/503. Nuclei were counterstained with DAPI (Thermo Fisher Scientific). Immunofluorescence images were acquired using a Leica SP8‐X confocal microscope, and the fluorescence integrated intensity quantified using Fiji/Image J Software.

### 2.8 Mitochondrial Reactive Oxygen Species (ROS) analysis

After 7 days of adipogenic differentiation, live cells were stained with 5 µM of Mitochondrial Superoxide Indicator, MitoSOX™ Red (Thermo Fisher Scientific) diluted in HBSS (Thermo Fisher Scientific) for 10 min at 37°C. Cells were then washed in HBSS for 10 min and fixed in 4% (vol/vol) PFA for 15 min at RT. Nuclei were stained with DAPI (Thermo Fisher Scientific). Immunofluorescence images were acquired and quantified as reported above.

### 2.9 Transmission Electron Microscopy analysis

Cells were detached with Trypsin 0.05% (Thermo Fisher) and resuspended in the Karnovsky fixative. After washing with 0.1 M phosphate buffer (pH 7.2), cells were post‐fixed in 1% osmium tetroxide (OsO4) for 90 min at RT, dehydrated by increasing concentration of alcohol and embedded in epoxy resin. 0.5 μm thick sections were stained with methylene blue and safranin. Subsequently, ultrathin 60–80 nm thick sections were collected on a 300‐mesh copper grid and, after staining with uranyl acetate and lead citrate, were qualitatively examined under a transmission electron microscope Philips EM 208S (Fei Electron Optics BV). All chemicals were purchased by Sigma‐Aldrich. High power micrographs collected at 5600× magnification were employed to evaluate the volume fraction occupied by lipid droplets. All morphometric data were blindly collected.

### 2.10 Immunofluorescence analysis of human heart tissue

Human ventricular samples were processed as previously described.^23^, ^24^ Briefly, they were fixed in 4% PFA (Santa‐Cruz) in PBS (Lonza) and processed for paraffin embedding. 6 μm thick sections were de‐waxed and rehydrated. Antigen retrieval was performed with Dako target retrieval solution citrate pH6 at 90°C. Human sections were incubated with anti‐GCN5 (1:100; Cell Signaling, #3305, rabbit), anti‐4HNE (1:200; Abcam, ab46545, rabbit) and anti‐cardiac Troponin T (1:300; ThermoFisher Scientific, #MA5‐12960, mouse) at 4°C overnight. After washing, sections were incubated with the appropriate fluorochrome‐conjugated secondary antibody Alexa 488 (A11034, 1:200) or Alexa 633 (A221126, 1:200) (AlexaFluor) for 1h at room temperature in the dark. Nuclear staining was performed by incubating sections with Hoechst 33342 (1:1000, ThermoFisher Scientific). Images were acquired with a confocal microscope (Zeiss LSM710—ConfoCor3 LSM, Zeiss) using the software Zen 2008 (Zeiss) and quantified with the software AxioVision Rel. 4.8.

### 2.11 Transcriptome analysis

Total RNA was extracted using Direct‐zol RNA Kit (Zymo Research), according to manufacturer's instructions. The concentration and purity of the isolated total RNA were assessed by Nanodrop and Qubit, while the integrity was evaluated using the Experion RNA StdSens Analysis Kit (Bio‐Rad). High‐quality RNA, with A_260/A280_ > 1.8, A_260/A230_ ranging from 1.8 up to 2.02 and RQI > 9.5, was used for subsequent library preparation. 500 ng of RNA for each sample was used for the preparations of the libraries, using the QuantSeq 3’ mRNA‐Seq Library Prep FWD (Lexogen). Samples were sequenced using the platform HiSeq2500, following the protocol SR1000. The results of the sequencing were collected in fastq files, using the software CASAVA, FastQC (Version 0.11.3). The reference genome was aligned to the Homo Sapiens assembly GRCh38 using STAR with recommended options and thresholds (version 2.5).^25^ HTSeq‐count was used to generate raw gene counts (version 0.9.). Subsequent analysis was performed in R (version 4.1.2). The data set was first pre‐filtered removing genes with an average read count per study group being lower than 64, which reduced the data set from 58,302 to 9284 genes. Annotation of genes was performed using the ensembldb^26^ package version 2.18.2 based on Ensembl release 90. Differential expression analysis was conducted using the DESeq2 package version 1.34.0^27^ employing a sex‐adjusted linear model. Resulting *p*‐values were adjusted for multiple hypothesis testing using the method from Benjamini and Hochberg. Genes with an adjusted *p*‐value smaller than 0.05 and an absolute log2 fold change larger than 0.7 (equivalent to a fold change of 1.62) were considered significant. Pathway enrichment analysis was performed using the EnrichmentBrowser Bioconductor package (version 2.24.0, method ‘ora’, *p*‐values adjusted using the method from Benjamini and Hochberg). The transcriptome data were deposited at the Gene Expression Omnibus (accession number GSE189657).

### 2.12 Droplet Digital PCR (ddPCR)

The validation of a subset of genes resulting differentially expressed from the transcriptome analysis was performed by ddPCR assays. A panel of 8 genes (*NAMPT*, *G6PD*, *GSR*, *ALDH2*, *ENO2*, *PGD*, *GYS1* and *ALDB1H1*) was selected among the genes with an adjusted *p*‐value < 0.05 and a log2FoldChange > |1|. Total RNA was extracted using Direct‐zol RNA Kit (Zymo Research) and reverse transcribed to cDNA using the SuperScript VILO cDNA Synthesis Kit (Invitrogen). The reaction mixture for the ddPCR (20 μl/reaction) contained 2× ddPCR Supermix for Probe (no dUTP) (Bio‐Rad), 20× primer/probe assay for each target (Table S1), 1 ng of cDNA and water up to the final volume. 20 μl of reaction was transferred with 70 μl of Droplet Generation oil for Probes (Bio‐Rad) in a DG8 Cartridge (Bio‐Rad). The cartridges were inserted into the QX200™ Droplet Generator (Bio‐Rad) to generate 40 μl droplet suspension then transferred into a 96‐well PCR plate (Bio‐Rad). The PCR reaction was performed using a GeneAmp™ PCR System 9700 (Applied Biosystems). The protocol conditions consisted of 95°C for 10 min, (94°C for 30 s, 60°C for 1 min) × 40 cycles, 98°C for 10 min and 4 °C for the storage. Amplification signals were read using the QX200™ Droplet Reader and analysed using the QuantaSoft software (Bio‐Rad).

### 2.13 Glutathione measurements

Reduced (GSH) and oxidized glutathione (GSSG) quantitation was performed as previously described.^28^ Cell pellet was resuspended in 50 µl of PBS, proteins were precipitated with 50 µl of 10% trichloroacetic acid plus 1 mM EDTA and, after a further dilution 1:5 with formic acid 0.1%, samples were analysed by LC‐MS/MS. The LC‐MS/MS analysis was performed using an Accela HPLC System (Thermo Fisher Scientific, Waltham, Massachusetts, USA) coupled to a triple quadrupole mass spectrometer TSQ Quantum Access (Thermo Fisher Scientific, Waltham, Massachusetts, USA) outfitted with electrospray ionization source operating in positive mode. The chromatographic separation was conducted on a Luna PFP column (2.0 × 100 mm, particle size 3.0 µm, Phenomenex, Torrance, California, USA) maintained at 35°C. Analytes were eluted under isocratic conditions at 200 µl/min by 1% methanol in 0.75 mM ammonium formate adjusted to pH 3.5 with formic acid. The analytes were detected by multiple reaction monitoring, and the transitions monitored (precursor ion > product‐fragment ions) were m/z 308.1 → m/z 76.2, 84.2, 161.9 (GSH) and m/z 613.2 → m/z 230.5, 234.6, 354.8 (GSSG). A linear 6‐point calibration curve (range 0.25–8 µM for GSH and 0.008–0.25 µM for GSSG) was used for the quantification.

### 2.14 Statistical analysis

Cells from a minimum of 3 independent individuals were included in each experiment. Cells obtained from different patients or from the same patient but in different amplification steps were considered as biological replicates. Details are given in the figure legend. Western Blot and ddPCR data are reported as median with interquartile range (IQR), and significance was assessed by non‐parametric Mann–Whitney test, setting alpha = 0.05, therefore considering evidence of significance if *p*‐value ˂ 0.05 (GraphPad Prism software 8.2.0). Quantification of immunostaining, visually described using median and interquartile range (IQR), was analysed fitting random intercept models (mixed or multilevel models), that account for the experimental hierarchical structure and the dependency between technical replicates, using the ‘mixed’ command implemented in Stata 16 (2019. Stata Statistical Software: Release 16. College Station, TX: StataCorp LLC. StataCorp.). The details on statistics and experimental N are reported in the figure legend of each specific experiment.

## 3 RESULTS

### 3.1 GCN5 protein expression is increased in ACM samples

**FIGURE 1:**
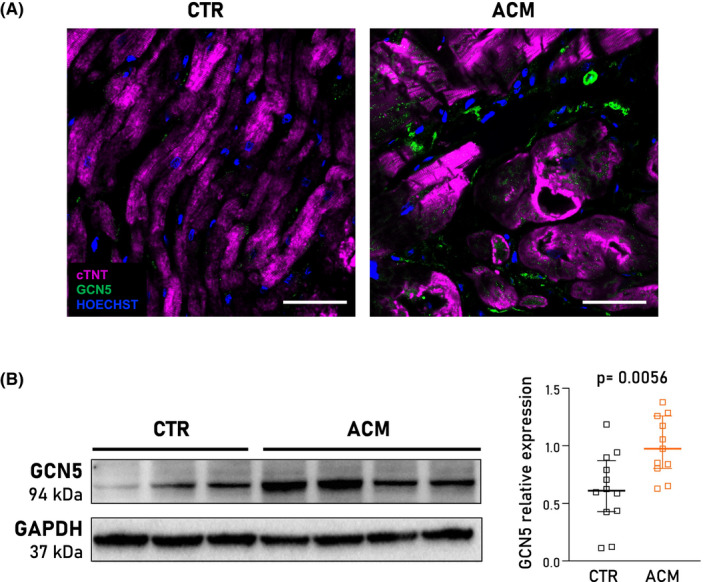
GCN5 expression in human right ventricle tissue sections and CStCs. (A) Representative immunofluorescence images of GCN5 expression (*green*) in human endomyocardial biopsies from one healthy control (CTR) and one ACM subject. Nuclei are counterstained with HOECHST (*blue*), myocardial tissue with troponin T (cTNT, *magenta*). Original Magnification 40×; scale bar 50 µm. (B) Western blot panels and densitometric analysis showing the GCN5 protein expression in CTR (*black*) and ACM (*orange*) CStCs cultured in basal medium. Mann–Whitney test, *p *= 0.0056 vs. CTR. Results are based on *N* = 7 independent CTR individuals with 12 replicates and *N* = 6 ACM patients with 11 replicates

Tissue distribution of GCN5 was investigated on endomyocardial biopsies of ACM patients and correspondent samples from cardiac tissue donors (CTR). Immunofluorescence analysis revealed that GCN5 was abundantly present in non‐cardiomyocyte cells in ACM samples, while its expression was barely detectable in CTR heart tissue (Figure 1A). Considering this finding and our prior evidence that CStCs are a source of adipocytes in ACM hearts,^7^ we next evaluated the expression of GCN5 *in vitro* in CStCs. Western blot analysis showed higher GCN5 protein level in ACM compared to CTR CStCs (Figure 1B).

### 3.2 Intracellular lipid accumulation is reduced upon cellular GCN5‐knockdown in ACM CStCs

**FIGURE 2:**
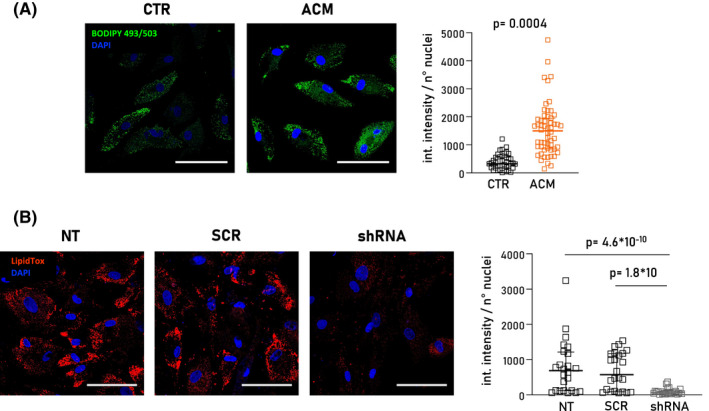
Analysis of intracellular lipid accumulation in CTR, ACM and GCN5‐knocked down ACM CStCs. (A) Representative immunofluorescence images of intracellular lipid droplets stained with BODIPY 493/503 (*green*) in CTR and ACM CStCs cultured in ADIPO for 7 days. Nuclei are counterstained with DAPI (*blue*). Original Magnification 40×; scale bar 100 µm. The scatter plot (*right panel*) shows the quantification of BODIPY 493/503 integrated intensity normalized on the total number of nuclei per field in CTR and ACM CStCs. Results are based on *N* = 5 independent CTR and *N* = 6 independent ACM with 5–15 available microscopy fields for each individual. Random intercept model, *p* = 0.0004 vs. CTR. (B) Representative immunofluorescence images showing intracellular lipid droplets stained with HCS LipidTOX™ (*red*) in not transduced (NT), transduced with scramble (SCR) or shRNA GCN5 ACM CStCs. Nuclei are counterstained with DAPI (*blue*). Original Magnification 40×, scale bar 100 µm. The scatter plot (*right panel*) shows the quantification of HCS LipidTOX™ integrated intensity normalized on the total number of nuclei. Results are based on *N* = 3 ACM‐independent patients with 8 available microscopy fields for each treatment group. Random intercept model, shRNA *p* = 4.6 × 10^−10^ vs NT; shRNA *p* = 1.8 × 10^−14^ vs SCR. Int. intensity: integrated intensity

Lipid substitution is one of the major phenotypical manifestations of ACM. To model adipogenesis *in vitro*, CStCs isolated from CTR and ACM individuals were exposed to ADIPO medium for 7 days, as previously described.^7^ We next investigated lipid accumulation in treated cells. As expected,^7^ ACM CStCs exposed to adipogenic medium (ADIPO) exhibited higher intracellular lipid accumulation than CTR cells as revealed by BODIPY 493/503 staining (Figure 2A).

To investigate the possible connection between the higher GCN5 expression and the increased lipid accumulation, we knocked down GCN5 in ACM CStCs. These cells were cultured in ADIPO medium and transduced with lentiviral particles encoding both shRNA GCN5 and GFP. As experimental controls, some cells did not get transduced (NT) and others were transduced with a scramble construct (SCR). After 7 days, transduction efficiency was estimated around 80% of the GFP‐positive cells over the total number of cells (Figure S1A). Western blot analysis confirmed approximatively a 50% reduction of GCN5 protein level in ACM CStCs exposed to shRNA GCN5 compared to not transduced and SCR transduced cells (Figure S1B). Importantly, GCN5 knockdown corresponded with a significant decrease in intracellular lipid accumulation in shRNA GCN5 compared with SCR and NT groups (Figure 2B). No difference was observed between NT and SCR samples.

### 3.3 Pharmacological GCN5 specific inhibition decreases intracellular lipid accumulation

**FIGURE 3:**
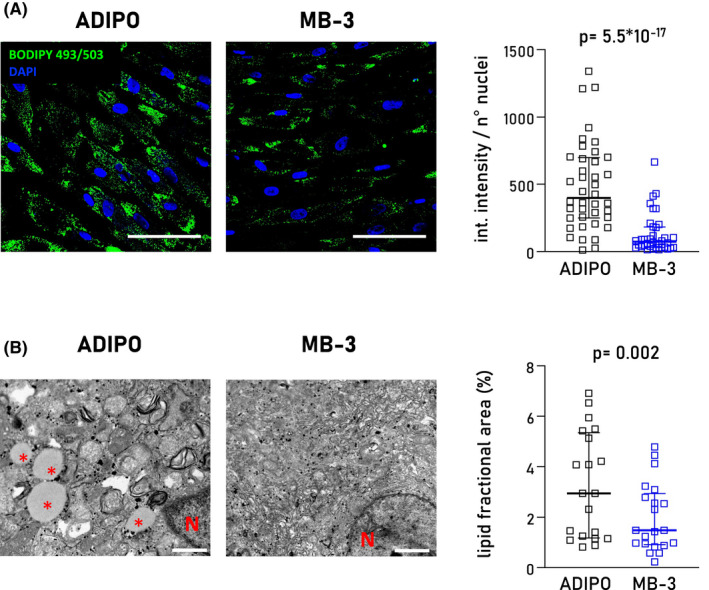
Effect of GCN5 inhibitor MB‐3 on intracellular lipid accumulation evaluated in ACM CStCs. (A) Representative immunofluorescence images of intracellular lipid droplets stained with BODIPY 493/503 (*green*) in ACM CStCs cultured in ADIPO medium for 7 days either in absence or in presence of MB‐3 (*blue*). Nuclei are counterstained with DAPI. Original Magnification 40×; scale bar 100 µm. The scatter plot (*right panel*) shows the quantification of BODIPY 493/503 integrated intensity normalized on the total number of nuclei per field in ACM CStCs either in absence or in presence of MB3. Results are based on *N* = 6 ACM‐independent patients with 5–15 microscopy fields available for the two treatment groups. Random intercept model, *p* = 5.5 × 10^−17^ vs ADIPO. (B) Representative transmission electron microscopy (TEM) images showing intracellular lipid droplets in ACM CStCs cultured in ADIPO medium, (original magnification 11,000×, 500 nm) and ADIPO in presence of MB‐3 (original magnification 5600×, 1000 nm). Red asterisks indicate lipid droplets, while the ‘N’ indicates cell nuclei. The scatter plot (right panel) shows the lipid fractional areas in the two different conditions tested in ACM patients. Results are based on *N* = 3 ACM‐independent patients with 5–9 microscopy fields available for the two treatment groups. Random intercept model, *p* = 0.002 vs ADIPO. Int. intensity: integrated intensity

As we demonstrated above, GCN5 downregulation leads to reduced lipid accumulation in ACM CStCs. We further investigated the effect of GCN5 pharmacological inhibition on intracellular lipid accumulation by exposing ACM CStCs to the specific GCN5 inhibitor MB‐3.^29^ Cells were cultured in ADIPO medium, and a second group was supplemented with 200 μM MB‐3 for 7 days. MB‐3 administration did not affect cell viability (Figure S2). Of note, the treatment with MB‐3 significantly reduced the lipid accumulation in ACM CStCs (Figure 3A). An independent evaluation of intracellular lipid accumulation in the presence or absence of MB‐3 was also performed by TEM analysis. ACM CStCs exposed to ADIPO supplemented with MB‐3 showed a significant reduction of the ratio between the area occupied by lipid droplets and the total area of the analysed cells (Figure 3B).

### 3.4 Transcriptome analysis of GCN5 inhibition reveals its role in the cellular oxidative pathways

**FIGURE 4:**
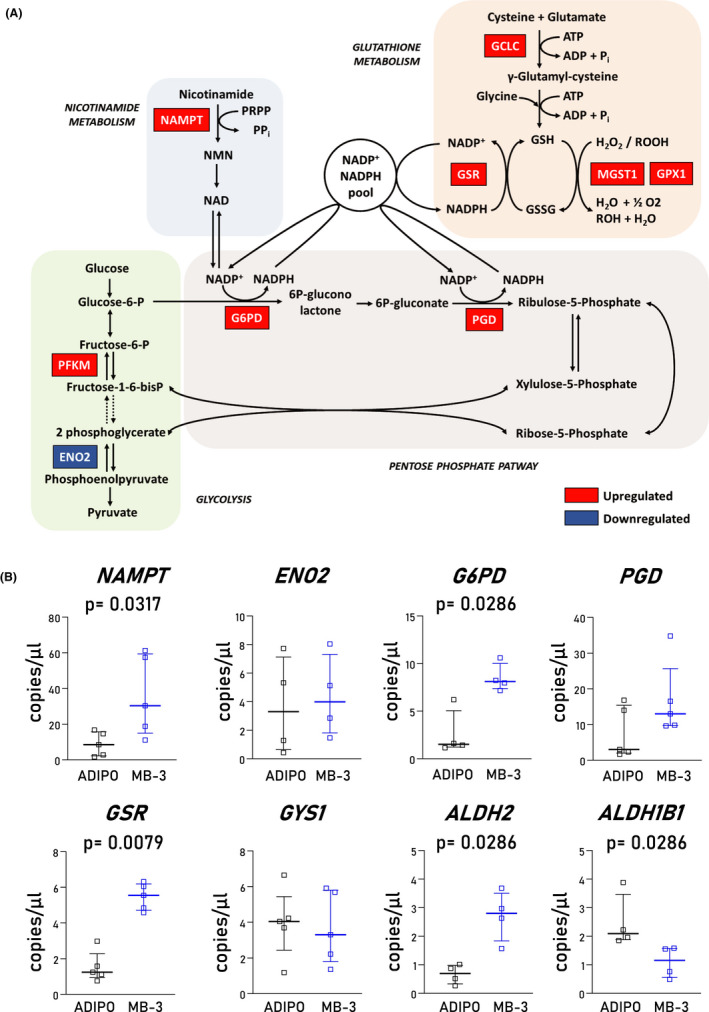
Gene expression analysis reveals that GCN5 inhibition implicates changes in the glutathione metabolism (A) Graphical representation of the genes related to the glutathione metabolism upregulated (*red*) and downregulated (*blue*) in ACM cells treated with MB‐3 and compared to ACM. (B) Validation by ddPCR of gene transcripts emerging from the transcriptome analysis. Scatter plots show copies per microliter of gene transcripts calculated by ddPCR analysis on ACM CStCs in absence or in presence of MB‐3. Results are based on *N* = 4 ACM‐independent patients, Mann–Whitney test, *NAMPT*, *p *= 0.0317; *ENO2*, ns, *p *= 0.685; *G6PD*, *p *= 0.0286; *PGD*, ns, *p *= 0.3095; *GSR*, *p *= 0.0079; *GYS1*, ns, *p* = 0.8413; *ALDH2*, *p *= 0.0286; *ALDHB1*, *p *= 0.0286 vs. CTR

We next performed a transcriptome analysis on ACM CStCs exposed to ADIPO medium for 7 days in presence or absence of MB‐3 to evaluate whether reduced lipid accumulation after GCN5 inhibition is mediated by cell metabolism regulation. In total, 502 genes were found to be regulated by MB‐3 treatment (Figure S3). These were enriched in 8 biological pathways (Table S2). Notably, the glutathione metabolism pathway resulted significantly affected by the MB‐3 treatment (Figure 4A). This prompted us to specifically investigate pathways related to cell metabolism and redox signalling. Table 2 shows the total size and the number of significant genes for each selected process. The differential expression of the single genes listed in Table 2 is reported in the supplementary file (Figures S4–S11; Tables S3–S10).

**TABLE 2:** List of manually selected KEGG pathways from the transcriptome analysis. Columns *Size* and *Count* contain the total number of genes of the selected pathway and the number of genes differentially expressed after MB‐3 treatment in ACM CStCs, respectively

ID	Name	Size	Count	Genes
hsa00480	Glutathione metabolism	34	8	*G6PD, GCLC, GCLM, GSR, GSTM3, IDH1, PGD, CHAC1*
hsa00600	Sphingolipid metabolism	28	5	*GLA, SMPD1, PLPP3, SPHK1, SPTLC2*
hsa00010	Glycolysis/Gluconeogenesis	40	6	*ADH1B, ENO2, ALDH2, ALDH1B1, ALDH3A2, PFKM*
hsa00071	Fatty acid degradation	28	4	*ADH1B, ALDH2, ALDH1B1, ALDH3A2*
hsa00030	Pentose phosphate pathway	18	3	*G6PD, PFKM, PGD*
hsa03320	PPAR signalling pathway	31	3	*ILK, ME1, PPARA*
hsa00760	Nicotinate and nicotinamide metabolism	23	1	*NAMPT*

To confirm the changes seen in the transcriptome analysis, the expression of eight genes with statistical evidence of differential expression was validated by ddPCR (Figure 4B). Among them, the genes *NAMPT*, *G6DP*, *GSR and ALDH2* were confirmed to be upregulated after MB‐3 treatment, while *ALDH1B1* was confirmed as downmodulated.

### 3.5 GCN5 decreased oxidative stress and acts on the glutathione metabolism

**FIGURE 5:**
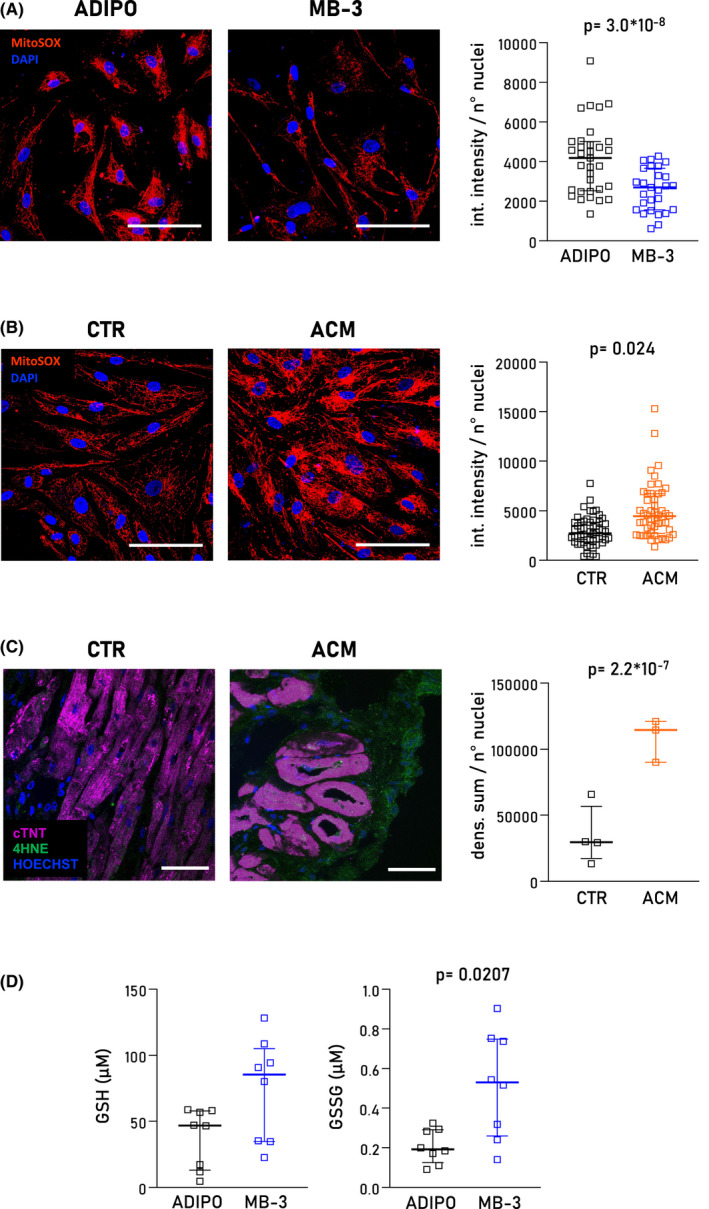
Effect of MB‐3 on mitochondrial ROS accumulation. (A) Representative immunofluorescence images of mitochondrial ROS stained with MitoSOX™ (*red*) in ACM CStCs after 7‐day exposure to ADIPO in absence or in presence of MB‐3. Nuclei are counterstained with DAPI (*blue*). Original Magnification 40×; scale bar 100 µm. The scatter plot (*right panel*) shows the quantification of MitoSOX™ integrated intensity normalized on the total number of nuclei per field in ACM CStCs in the absence or presence of MB‐3. Results are based on *N* = 4 ACM‐independent patients, with 5–9 microscopy fields available for the two treatment groups. Random intercept model, *p* = 3.0 × 10^−8^ vs ADIPO. (B) Representative immunofluorescence images of mitochondrial ROS stained with MitoSOX™ in CTR CStCs and ACM CStCs exposed to ADIPO for 7 days. Nuclei are counterstained with DAPI. Original Magnification 40×; scale bar 100 µm. On the right panel, scatter plot showing the quantification of MitoSOX™ integrated density normalized on the total number of nuclei. Results are based on *N* = 6 CTR individuals and *N* = 5 ACM patients, with 5–14 microscopy field per sample. Random intercept model *p *= 0.024 vs CTR. (C) Representative immunofluorescence images of human cardiac tissue stained with 4HNE (*green*) from one healthy control (CTR) and one ACM subject. Nuclei are counterstained with HOECHST (*blue*), myocardial tissue with troponin T (cTNT, *magenta*). The densitometric analysis (*right panel*) shows the quantification of the 4HNE normalized on the nuclei number. Results are based on *N* = 3 ACM patients and *N* = 4 CTR, with 4–5 microscopy field for each individual. Random intercept model *p* = 2.27 × 10^−7^ (D) Effect of MB‐3 on cellular glutathione system. Levels of reduced (GSH) and oxidized glutathione (GSSG) were assessed on cell lysates. Results are based on *N* = 8 ACM patients. Mann–Whitney test, GSH, ns *p *= 0.08; GSSG *p *= 0.0207 vs. ADIPO. Int. intensity: integrated intensity; dens. Sum: densitometric sum

Considering the impact of MB‐3 treatment on cellular redox pathways proven by transcriptome analysis, we next evaluated the accumulation of reactive oxygen species (ROS) in the cells. We compared fluorescence intensity of MitoSOX™, a specific mitochondrial superoxide indicator, between ACM CStCs exposed to ADIPO medium and ACM CStCs exposed to ADIPO medium supplemented with MB‐3 for 7 days. MB‐3 treatment led to a reduction in mitochondrial ROS production (Figure 5A). In addition, MitoSOX fluorescence intensity was higher in ACM compared to CTR CStCs in ADIPO medium (Figure 5B). In agreement, oxidative stress measured by 4HNE staining resulted higher in heart tissues from ACM patients compared to CTR donors, and particularly evident in non‐cardiomyocyte cells (Figure 5C). Of note, the treatment of ACM CStCs with the mitochondrial scavenger MitoTempo was able not only to reduce mitochondrial oxidative stress but also the intracellular accumulation of lipid droplets (Figure S12). However, mitochondrial ultrastructure and mitochondrial network (Figure S13) showed no difference in ACM vs CTR CStCs cultured for 7 days in ADIPO medium.

Finally, to confirm the link between the modulation of ROS and GCN5 inhibition highlighted by the transcriptome analysis, we assessed cellular levels of both reduced (GSH) and oxidized glutathione (GSSG). MB‐3‐treated ACM CStCS presented an upregulated glutathione metabolism increasing both GSH and GSSG (Figure 5D), suggesting a role for MB‐3 in the induction of detoxifying processes.

## 4 DISCUSSION

Arrhythmogenic cardiomyopathy (ACM) is a cardiac genetic disease hallmarked by ventricular arrhythmias, progressive myocardial fibro‐adipose replacement, heart failure and sudden death.^30^ ACM is mainly caused by pathogenic variants in genes encoding desmosomal proteins, with *PKP2* being the most common causal gene (7–51%), followed by *DSP* (1–16%) and *DSG2* (5–25%), whereas causal variants in *DSC2* and *JUP* are rarer.^31^ Although its genetic basis^32^ has been thoroughly studied and the disease diagnosis has advanced, ACM appears as a multifaceted disease in which the pathogenic molecular mechanisms are still poorly understood. Environmental factors,^33^ as in particular intense exercise,^34^ have been shown to play a role as phenotypic modulators, while much less is known about the relevance of epigenetic regulators in ACM pathophysiology.^35^

Over the last ten years, several works have demonstrated that different epigenetics enzymes such as histone acetyltransferases (HATs) and deacetylase (HDAC) play an important role not only in governing gene expression changes underlying cardiac diseases,^10^ but also in regulating cell energy metabolism, affecting enzymes of glycolysis, glucose oxidation, electron transport chain and fatty acid β‐oxidation.^11^ In the present work, we observed abundant expression of the acetyltransferase GCN5 in non‐cardiomyocyte cells from endomyocardial biopsies of ACM patients compared to healthy control donors. We then used human cardiac stromal cells (CStCs) obtained from ACM heart biopsies as *in vitro* model, since we previously demonstrated they represent a reliable cell model to study ACM pathogenesis.^7^, ^8^ In agreement with the increased levels of GCN5 found in pathological human heart specimens, ACM CStCs showed higher GCN5 protein level compared to CTR CStCs. In addition, when exposed to the adipogenic (ADIPO) medium, ACM cells showed higher lipid accumulation compared to CTR cells. Interestingly, when GCN5 was knocked down in ACM CStCs exposed to ADIPO medium, a significant decrease in intracellular lipid accumulation was observed in comparison with scramble cells.

Of note, a link between adipogenesis and the acetyltransferase GCN5 has already been reported.^36^, ^37^ The HAT family, to which GCN5 belongs, is known to be involved in adipogenesis regulation, increasing mRNA level of adipogenic markers, such as adiponectin, FABP4, C/EBPα and PPARγ.^38^ Wiper‐Bergeron and colleagues identified GCN5 as a crucial effector of C/EBPβ acetylation in NIH 3T3 and 3T3 L1 cells, enhancing C/EBPβ‐directed transcription and potentiating C/EBPβ‐dependent preadipocyte differentiation.^36^ Further, our findings also agree with the report of Jin and collaborators showing that immortalized brown preadipocytes double knocked out for GCN5/PCAF and exposed to an external adipogenic stimulus had a reduced expression of adipogenic genes and lipid droplet accumulation.^37^

Current treatment options for ACM are based on symptom management and prevention, such as anti‐arrhythmic drugs, ICD implantation and, ultimately, heart transplantation, but no pharmacological approaches are available in the clinical practice to counteract cardiac adipogenic substitution. As such, GCN5 appears as an interesting pharmacologically target to possibly reduce lipid accumulation and disease severity. Academic and pharmaceutical companies have started to work on the development of new therapeutic options termed ‘epigenetic therapy’, because many human diseases display an epigenetic aetiology. Indeed, several small molecule that inhibit GCN5 (i.e. MB‐3) have been discovered and already taken into consideration for the treatment of different pathologies associated with GCN5 dysregulation like type 2 diabetes, insulin resistance, metabolic disease and syndrome, dyslipidaemia, obesity or overweight, neurodegenerative diseases, heart failure, muscle diseases and improvement of exercise endurance capacity.^39^, ^40^

Therefore, the MB‐3 compound was tested in the ACM context. ACM CStCs supplemented with MB‐3 showed a significant reduction in lipid accumulation, demonstrated both by IF and TEM analysis. A subsequent transcriptome analysis revealed the modulation of different genes linked to energetic metabolism and to redox pathways in MB‐3‐treated CStCs. Interestingly, we observed that the expression level of integrin‐linked kinase (*ILK*), a novel gene recently linked to ACM, was lower in ACM CStCs after exposure to MB‐3. ILK is a serine/threonine protein kinase associated with various pathways of cardiac remodelling^41^ and intracellular signalling as adipogenic differentiation.^42^ Muscle‐specific *Ilk* transgenic mice showed the development of an ACM phenotype.^43^ Of note, transgenic zebrafish with a cardiac‐specific overexpression of human *ILK* variants found in ACM patients developed cardiac dysfunction and severe epicardial fat tissue,^41^ thus highlighting the potential role of MB‐3 in rescuing the adipogenic phenotype. This is also in line with a recent work showing that ILK knockout in mice and 3T3‐L1 cells decreased lipid accumulation and CD36 gene expression during adipogenesis.^42^ Specifically, nicotinamide phosphoribosyltransferase (NAMPT), the rate‐limiting enzyme that allows the biosynthesis of NAD^+^,^44^ was upregulated. It has been suggested that increased levels of NAD^+^ via NAMPT could represent a protective strategy against the effects of metabolic disease.^44^ The exposure of ACM CStCs to MB‐3 led to the upregulation of other genes also related to the redox balance, such as glucose‐6‐phosphate dehydrogenase (*G6PD*), glutathione reductase (*GSR*) and aldehyde dehydrogenase (*ALDH2*). G6PD is an enzyme involved in the reduction of NADP to NADPH via the pentose phosphate pathway (PPP).^45^ NADPH is an important metabolite implicated in the maintenance of cellular antioxidant capacities.^45^ Along with that, GSR is responsible for keeping the glutathione (GSH) pool in the reduced form.^46^ As such, GSH can ensure cellular control of the ROS. Altogether, MB‐3 treatment upregulates glutathione metabolism to respond to alteration of redox balance. Similarly, ALDH2 plays an important protective role at mitochondrial level in several human diseases (neurodegenerative diseases, stroke, cancer), including heart failure and cardiac dysfunction triggered by ischaemic injury, hypertension, alcohol and diabetes.^47^ ALDH2 also plays a key role in improving detoxification of reactive aldehydic products arising from lipid peroxidation under oxidative stress, such as 4‐hydroxy‐2‐nonenal (4HNE) and malondialdehyde (MDA), thus exerting a protective effect against acute (ischaemia) and chronic (heart failure) cardiovascular diseases.^48^, ^49^ Of note, the upregulation of ALDH2 mediated by MB‐3 treatment and following reduction of lipid peroxidation is particularly intriguing, considering the higher 4HNE expression observed in heart tissues from ACM patients compared to CTR donors. Along with that, ACM CStCs exposed to ADIPO medium also showed increased mitochondrial ROS compared to CTR CStCs.

In summary, our results indicate that GCN5 pharmacological inhibition brings to reduced intracellular lipid accumulation by acting on different targets associated not only with lipid metabolism but also to redox processes. This observation agrees with recent literature highlighting the contribution of ROS to adipocyte differentiation in mesenchymal stromal cells.^50^, ^51^, ^52^ Also, our recent report showed that ACM patients are characterized by high plasma levels of oxLDL and by cardiac lipid peroxidation, suggesting that the oxidative environment is a strong determinant of the ACM adipogenic phenotype and severity.^8^ The key role of oxidative stress in ACM pathophysiology is confirmed in the present study by the fact that ACM patient biopsies demonstrate higher amount of oxidative stress measured by 4HNE staining compared to CTR donors. Of note, it has been recently demonstrated that lipid peroxidation metabolites, as 4HNE, may activate a robust intracellular Ca^2^ ^+^ influx through transient receptor potential A1 (TRPA1) channels in the endothelium of cerebral arteries,^53^, ^54^ thus suggesting that oxidative stress acting on Ca^2+^ signalling trough TRP channels might represent an intriguing mechanism involved in the dysfunction of ACM tissues and differentiation of CStCs.

Further, MB‐3 treatment of ACM CStCs seemed to protect from mitochondrial ROS production, as demonstrated by decreased MitoSOX fluorescence intensity, thus highlighting a possible specific functional role for mitochondria in the disease.^55^ Notably, MB‐3 also boosted cellular detoxification systems by acting on glutathione homeostasis slightly increasing reduced glutathione (GSH) and significantly increasing oxidized glutathione (GSSG) levels. In agreement, Costantino et al. reported that GCN5 can epigenetically regulate oxidant and ROS scavenger enzymes, though its inhibition might prevent the increase in oxygen radicals in human endothelial cells.^56^ In addition, it has been already reported a link between acetylation mediated by GCN5 and the modulation of the cellular response to oxidative stress in yeast.^57^

Of note, GCN5 has many molecular targets; for instance, it has been shown that GCN5 directly acetylates and inhibits the activity of proliferator‐activated receptor gamma coactivator 1‐alpha (PGC1‐α), a master regulator of mitochondrial biogenesis, mitophagy and cellular energy metabolism.^16^, ^58^ Further, GCN5 promotes the acetylation of mitochondrial fatty acid oxidation enzymes thus regulating cardiac^59^ and hepatic^60^ metabolic homeostasis. In Drosophila and mammalian cells, GCN5 inhibits autophagy and lysosome biogenesis by targeting TFEB, the master transcription factor for autophagy‐ and lysosome‐related gene expression.^61^ Several studies also reported GCN5 as negative regulator of inflammatory and immunity response by suppressing NF‐κB transcriptional activity^62^ and interferon‐β production,^63^ respectively. Therefore, considering the complex and pleiotropic role of GCN5, we can conceive that the reduced lipid accumulation observed after its inhibition may partly depend on other mechanisms than the cellular redox. However, the investigation of these other mechanisms is beyond the scope of this manuscript and other studies are needed to further investigate the issue.

In conclusion, the present work describes a link between ACM pathophysiology and the histone acetyltransferase GCN5, demonstrating its contribution to lipid accumulation and oxidative stress. GCN5 silencing or pharmacological inhibition results in a reduced intracellular fat accumulation, and a modulation of the cellular redox processes. Our findings provide a novel pharmacological target and could potentially open new therapeutic perspectives to mitigate the adipogenic phenotype associated with ACM.

## AUTHOR CONTRIBUTION

**Chiara Volani:** Data curation (equal); Formal analysis (equal); Investigation (equal); Methodology (equal); Visualization (equal); Writing – original draft (equal); Writing – review & editing (equal). **Alessandra Pagliaro:** Data curation (equal); Formal analysis (equal); Investigation (equal); Methodology (equal); Writing – review & editing (equal). **Johannes Rainer:** Formal analysis (equal); Methodology (equal); Software (equal); Visualization (equal); Writing – review & editing (equal). **Giuseppe Paglia:** Visualization (equal); Writing – original draft (equal); Writing – review & editing (equal). **Benedetta Porro:** Methodology (equal); Writing – review & editing (equal). **Ilaria Stadiotti:** Methodology (equal); Writing – review & editing (equal). **Luisa Foco:** Data curation (equal); Methodology (equal); Writing – review & editing (equal). **Elisa Cogliati:** Resources (equal). **Adolfo Paolin:** Resources (equal). **Costanza Lagrasta:** Data curation (equal); Methodology (equal); Writing – review & editing (equal). **Caterina Frati:** Data curation (equal); Methodology (equal); Writing – review & editing (equal). **Emilia Corradini:** Methodology (equal). **Angela Falco:** Methodology (equal). **Theresa Matzinger:** Investigation (equal); Methodology (equal). **Anne Picard:** Methodology (equal). **Benedetta Ermon:** Methodology (equal). **Silvano Piazza:** Methodology (equal); Software (equal). **Marzia De Bortoli:** Methodology (equal); Supervision (equal); Writing – review & editing (equal). **Claudio Tondo:** Resources (equal). **Philippe Reginald:** Investigation (equal); Methodology (equal). **Medici Andrea:** Investigation (equal); Methodology (equal). **Lavdas A. Alexandros:** Methodology (equal). **Michael J.F. Blumer:** Investigation (equal); Methodology (equal). **Giulio Pompilio:** Funding acquisition (equal); Resources (equal); Writing – review & editing (equal). **Elena Sommariva:** Data curation (equal); Investigation (equal); Resources (equal); Writing – review & editing (equal). **Peter Pramstaller:** Funding acquisition (equal); Writing – review & editing (equal). **Troppmair Jakob:** Conceptualization (equal); Funding acquisition (equal); Supervision (equal). **Viviana Meraviglia:** Conceptualization (equal); Data curation (equal); Investigation (equal); Supervision (equal); Writing – original draft (equal); Writing – review & editing (equal). **Alessandra Rossini:** Conceptualization (equal); Funding acquisition (equal); Project administration (equal); Supervision (equal); Writing – original draft (equal); Writing – review & editing (equal).

## CONFLICTS OF INTEREST

The authors declare no conflict of interest.

## Supporting information

Supplementary Material

## Data Availability

The data contained used to support the findings of this study are available from the corresponding author upon request. The transcriptome analysis dataset supporting the conclusions of this article is available at the Gene Expression Omnibus, accession number GSE189657.
