# Supplementary material for: GCN5 contributes to intracellular lipid accumulation in human primary cardiac stromal cells from patients affected by Arrhythmogenic cardiomyopathy
Source: J Cell Mol Med. 2022 Jun 16;26(13):3687–701. doi: 10.1111/jcmm.17396 (PMC9258704; doi:10.1111/jcmm.17396)
Supplement: Supplementary file 1 — Supplementary Material [file JCMM-26-3687-s001.docx]

**SUPPLEMENTARY MATERIAL FOR:**

**GCN5 contributes to intracellular lipid accumulation in human primary cardiac stromal cells from patients affected by Arrhythmogenic Cardiomyopathy**

Volani C*, Pagliaro A* et al.

Correspondence to Alessandra Rossini: [alessandra.rossini@eurac.edu](mailto:alessandra.rossini@eurac.edu)

**Materials and Methods**

**Mitochondrial network analysis**

After 7 days of adipogenic differentiation, cells were fixed in 4% (vol/vol) PFA for 10 min at RT, permeabilized with 0.1% Triton X-100 in PBS, then incubated with anti-Grp75 (1:5000; ab53098, Abcam) at 4°C overnight. After washing, cells were incubated with the secondary antibody Alexa 555 (1:1000; A21429, Thermo Fisher Scientific) for 1h at room temperature in the dark. Nuclei were stained with DAPI (Thermo Fisher Scientific). Immunofluorescence images were acquired using a Leica SP8-X confocal microscope. The mitochondrial network was computationally reconstructed in three dimensions (Imaris software, Biplane, Zurich) and the total length as well as the number of unconnected parts of the network was measured. The ratio of the total length to the number of unconnected parts was used as a measure of fragmentation.

**MitoTEMPO treatment**

ACM CStCs treatment with the specific mitochondria Reactive Oxygen Species (ROS) scavenger Mitotempo (500 nM, Sigma-Aldrich) was performed in ADIPO medium for 7 days.

**Table S1**: List of digital droplet PCR assays.

| **Symbol** | **Aliases** | **Gene description** | **species** | **company** | **catalog nr set** | **Amplicon length** | **probe** |
| --- | --- | --- | --- | --- | --- | --- | --- |
| *ALDH2* | *ALDH2, ALDH-E2, ALDHI, ALDM* | aldehyde dehydrogenase 2 family (mitochondrial) | human | BioRad | dHsaCPE99778273 | 118 | FAM |
| *GYS1* | *GSY, GYS, GYS1* | glycogen synthase 1 | human | BioRad | dHsaCPE5057258 | 130 | FAM |
| *ENO2* | *ENO2, HEL-S-279, NSE* | enolase 2 | human | BioRad | dHsaCPE5025456 | 100 | FAM |
| *ALDH1B1* | *ALDH1B1, ALDH5, ALDHX* | aldehyde dehydrogenase 1 family member B1 | human | BioRad | dHsaCPE5052266 | 188 | FAM |
| *PGD* | *6PGD, PGD* | phosphogluconate dehydrogenase | human | BioRad | dHsaCPE5037612 | 92 | FAM |
| *NAMPT* | *1110035O14Rik, NAMPT, PBEF, PBEF1, VF, VISFATIN* | nicotinamide phosphoribosyltransferase | human | BioRad | dHsaEG5189065 | 104 | FAM |
| *GSR* | *GSR, HEL-75, HEL-S-122m* | glutathione-disulfide reductase | human | BioRad | dHsaCPE5053615 | 124 | FAM |
| *G6PD* | *G6PD, G6PD1* | glucose-6-phosphate dehydrogenase | human | BioRad | dHsaCPE5027925 | 137 | FAM |

**Figure S1:**

**Figure S1:** (**A**) Evaluation of shRNA GCN5 transduction efficiency in ACM CStCs. Representative images of ACM CStCs not transduced (NT), transduced with scramble (SCR) or with GCN5 shRNA. Original Magnification 4X; scale bar 200 µm**.** On the right panel, scatter plot displaying the percentage of transduced cells evaluated by GFP fluorescence. Results are based on: SCR, N=6 obtained from 3 independent ACM patients; shRNA, N=6 obtained from 3 independent ACM patients. Mann-Whitney test, shRNA vs. SCR, ns, p= 0.310. (**B**) Evaluation of GCN5 relative expression in ACM CStCs either NT, transduced with SCR or with the shRNA. Results are based on N=3 independent ACM patients. Kruskal-Wallis test, p= 0.05; post hoc comparison SCR vs shRNA: ns.

**Figure S2:**

**Figure S2:** Evaluation of cell viability of ACM CStCs after the treatment with MB-3. Scatter plot showing the percentage of live cells (evaluated as Trypan blue negative cells) after 7-day exposure to the ADIPO in the absence or presence of MB-3. Results are based on N=3 independent ACM patients. Mann Whitney test, ns, p= 0.5.

**Figure S3:**


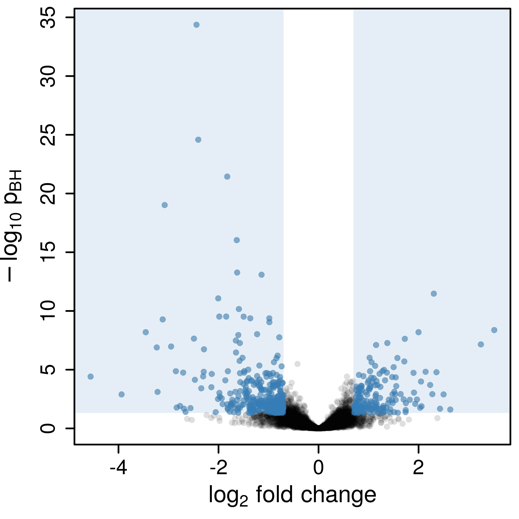


**Figure S3**: Volcano plot representing the overall differential expression of genes regulated by MB-3. In total 502 genes were found to be significantly impacted by the MB-3 treatment.

**Table S2**:

| **Pathway** | **Size** | **Count** | **p_adj_** |
| --- | --- | --- | --- |
| ECM-receptor_interaction | 42 | 14 | 3.53e-06 |
| Protein digestion and absorption | 36 | 13 | 3.53e-06 |
| Focal adhesion | 135 | 25 | 4.45e-06 |
| Amoebiasis | 53 | 14 | 0.00028 |
| Proteoglycans_in_cancer | 136 | 18 | 0.01813 |
| Glutathione metabolism | 34 | 8 | 0.01813 |
| Vascular_smooth_muscle_contraction | 61 | 11 | 0.01813 |
| Ferroptosis | 31 | 7 | 0.04741 |

**Table S2**: KEGG Pathways enriched with genes significantly regulated by MB-3 treatment in ACM cells. Columns *Size* and *Count* contain the number of genes quantified in the present data set associated to the pathway and the number of genes being significantly regulated by MB-3, respectively. Column *p_adj_*: adjusted enrichment p-value.

**Figure S4:**


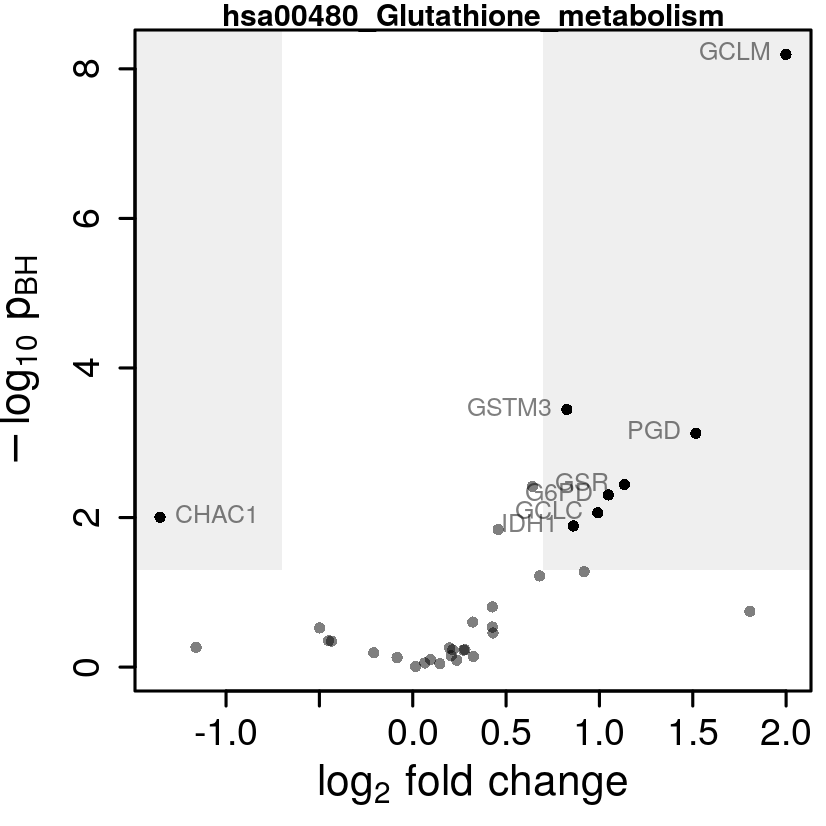


**Figure S4**: Volcano plot representing differential expression of genes involved in the Glutathione metabolism pathway. Genes within the grey shaded rectangles are considered significant.

**Table S3**:

| **Gene ID** | **Gene name** | **M** | **p_adj_** |
| --- | --- | --- | --- |
| ENSG00000023909 | *GCLM* | 2.00 | <0.00001 |
| ENSG00000134202 | *GSTM3* | 0.83 | 0.0004 |
| ENSG00000142657 | *PGD* | 1.52 | 0.0007 |
| ENSG00000104687 | *GSR* | 1.13 | 0.0036 |
| ENSG00000233276 | *GPX1* | 0.64 | 0.0038 |
| ENSG00000160211 | *G6PD* | 1.05 | 0.0050 |
| ENSG00000001084 | *GCLC* | 0.99 | 0.0086 |
| ENSG00000128965 | *CHAC1* | -1.35 | 0.0099 |
| ENSG00000138413 | *IDH1* | 0.86 | 0.0130 |
| ENSG00000100983 | *GSS* | 0.46 | 0.0144 |
| ENSG00000008394 | *MGST1* | 0.92 | 0.0530 |
| ENSG00000085871 | *MGST2* | 0.68 | 0.0604 |
| ENSG00000048392 | *RRM2B* | 0.43 | 0.1567 |
| ENSG00000211445 | *GPX3* | 1.81 | 0.1798 |
| ENSG00000164294 | *GPX8* | 0.32 | 0.2498 |
| ENSG00000148834 | *GSTO1* | 0.43 | 0.2911 |
| ENSG00000182054 | *IDH2* | -0.50 | 0.2993 |
| ENSG00000170899 | *GSTA4* | 0.43 | 0.3495 |
| ENSG00000116157 | *GPX7* | -0.45 | 0.4442 |
| ENSG00000102172 | *SMS* | -0.44 | 0.4499 |
| ENSG00000099998 | *GGT5* | -1.16 | 0.5457 |
| ENSG00000006625 | *GGCT* | 0.20 | 0.5534 |
| ENSG00000117592 | *PRDX6* | 0.28 | 0.5862 |
| ENSG00000002549 | *LAP3* | 0.21 | 0.5885 |
| ENSG00000197448 | *GSTK1* | 0.27 | 0.5915 |
| ENSG00000115365 | *LANCL1* | -0.21 | 0.6414 |
| ENSG00000167468 | *GPX4* | 0.21 | 0.7034 |
| ENSG00000143198 | *MGST3* | 0.32 | 0.7201 |
| ENSG00000167325 | *RRM1* | -0.08 | 0.7455 |
| ENSG00000117862 | *TXNDC12* | 0.10 | 0.7959 |
| ENSG00000115758 | *ODC1* | 0.24 | 0.8091 |
| ENSG00000084207 | *GSTP1* | 0.07 | 0.8804 |
| ENSG00000166825 | *ANPEP* | 0.15 | 0.9015 |

**Table S3**: Differential expression of genes involved in Glutathione metabolism. Columns *M* and *p_adj_* contain the log_2_ fold change and adjusted p-values, respectively.

**Figure S5:**


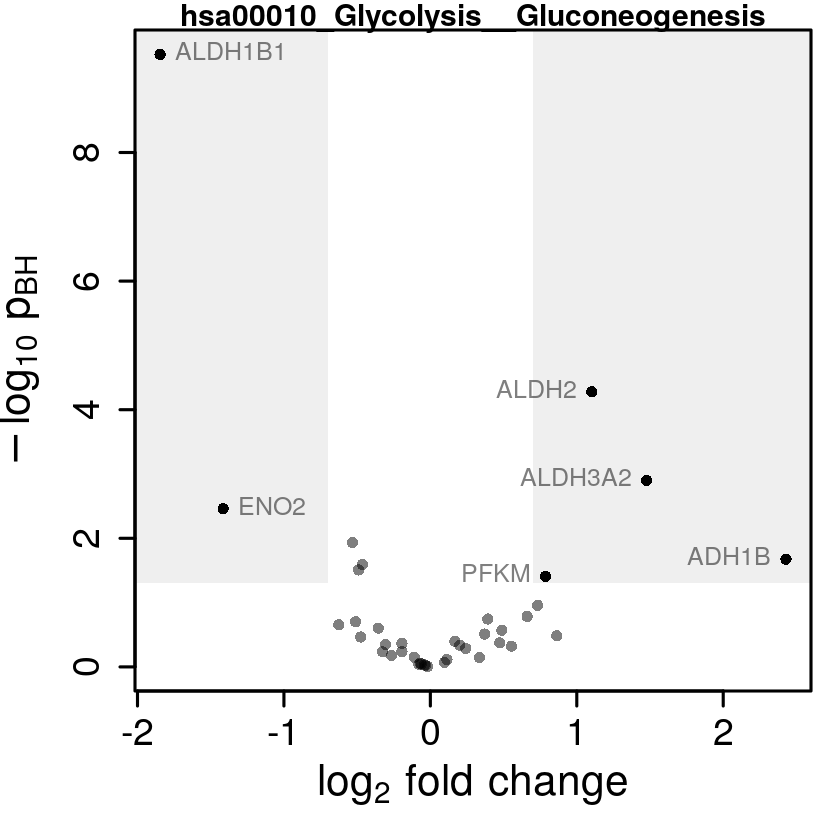


**Figure S5:** Volcano plot representing differential expression of genes involved in the Glycolysis/Gluconeogenesis pathway. Genes within the grey shaded rectangles are considered significant.

**Table S4:**

| **Gene ID** | **Gene name** | **M** | **p_adj_** |
| --- | --- | --- | --- |
| ENSG00000137124 | *ALDH1B1* | -1.85 | <0.00001 |
| ENSG00000111275 | *ALDH2* | 1.10 | 0.0001 |
| ENSG00000072210 | *ALDH3A2* | 1.48 | 0.0013 |
| ENSG00000111674 | *ENO2* | -1.41 | 0.0035 |
| ENSG00000111640 | *GAPDH* | -0.53 | 0.0116 |
| ENSG00000196616 | *ADH1B* | 2.43 | 0.0211 |
| ENSG00000141959 | *PFKL* | -0.46 | 0.0256 |
| ENSG00000105220 | *GPI* | -0.49 | 0.0310 |
| ENSG00000152556 | *PFKM* | 0.79 | 0.0391 |
| ENSG00000143891 | *GALM* | 0.73 | 0.1102 |
| ENSG00000143149 | *ALDH9A1* | 0.66 | 0.1640 |
| ENSG00000141349 | *G6PC3* | 0.39 | 0.1800 |
| ENSG00000067057 | *PFKP* | -0.51 | 0.1984 |
| ENSG00000134333 | *LDHA* | -0.63 | 0.2210 |
| ENSG00000168291 | *PDHB* | -0.36 | 0.2493 |
| ENSG00000111716 | *LDHB* | 0.49 | 0.2683 |
| ENSG00000117448 | *AKR1A1* | 0.37 | 0.3081 |
| ENSG00000154930 | *ACSS1* | 0.86 | 0.3274 |
| ENSG00000079739 | *PGM1* | -0.47 | 0.3424 |
| ENSG00000156515 | *HK1* | 0.17 | 0.3984 |
| ENSG00000109107 | *ALDOC* | 0.47 | 0.4222 |
| ENSG00000149925 | *ALDOA* | -0.19 | 0.4305 |
| ENSG00000074800 | *ENO1* | -0.31 | 0.4477 |
| ENSG00000131828 | *PDHA1* | 0.20 | 0.4600 |
| ENSG00000159399 | *HK2* | 0.55 | 0.4764 |
| ENSG00000067225 | *PKM* | 0.24 | 0.5153 |
| ENSG00000150768 | *DLAT* | -0.19 | 0.5782 |
| ENSG00000102144 | *PGK1* | -0.33 | 0.5816 |
| ENSG00000100889 | *PCK2* | -0.27 | 0.6631 |
| ENSG00000131069 | *ACSS2* | -0.11 | 0.7085 |
| ENSG00000006534 | *ALDH3B1* | 0.34 | 0.7158 |
| ENSG00000197894 | *ADH5* | 0.11 | 0.7677 |
| ENSG00000111669 | *TPI1* | 0.10 | 0.8492 |
| ENSG00000107789 | *MINPP1* | -0.07 | 0.8801 |
| ENSG00000169299 | *PGM2* | -0.06 | 0.8998 |
| **Gene ID** | **Gene name** | **M** | **p_adj_** |
| ENSG00000172331 | *BPGM* | -0.08 | 0.9093 |
| ENSG00000164904 | *ALDH7A1* | -0.04 | 0.9328 |
| ENSG00000091140 | *DLD* | -0.03 | 0.9494 |
| ENSG00000159322 | *ADPGK* | -0.02 | 0.9859 |

**Table S4:** Differential expression of genes involved in Glycolysis/Gluconeogenesis. Columns *M* and *p_adj_* contain the log_2_ fold change and adjusted p-values, respectively.

**Figure S6:**


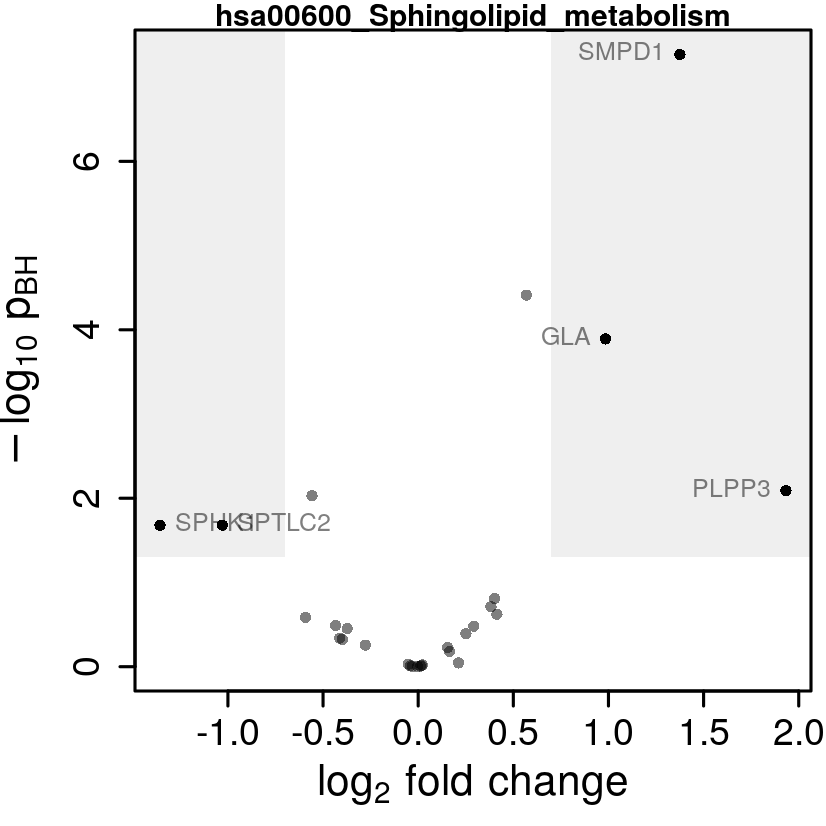


**Figure S6:** Volcano plot representing differential expression of genes involved in the Sphingolipid metabolism pathway. Genes within the grey shaded rectangles are considered significant.

**Table S5:** Differential expression of genes involved in Sphingolipid metabolism. Columns *M* and *p_adj_* contain the log_2_ fold change and adjusted p-values, respectively.

| **Gene ID** | **Gene name** | **M** | **p_adj_** |
| --- | --- | --- | --- |
| ENSG00000166311 | *SMPD1* | 1.38 | <0.00001 |
| ENSG00000197746 | *PSAP* | 0.57 | <0.00001 |
| ENSG00000102393 | *GLA* | 0.98 | 0.0001 |
| ENSG00000162407 | *PLPP3* | 1.93 | 0.0081 |
| ENSG00000198964 | *SGMS1* | -0.56 | 0.0093 |
| ENSG00000100596 | *SPTLC2* | -1.03 | 0.0209 |
| ENSG00000176170 | *SPHK1* | -1.36 | 0.0209 |
| ENSG00000104763 | *ASAH1* | 0.40 | 0.1552 |
| ENSG00000204386 | *NEU1* | 0.38 | 0.1943 |
| ENSG00000078124 | *ACER3* | 0.41 | 0.2378 |
| ENSG00000164023 | *SGMS2* | -0.59 | 0.2593 |
| ENSG00000100299 | *ARSA* | -0.43 | 0.3236 |
| ENSG00000170266 | *GLB1* | 0.29 | 0.3309 |
| ENSG00000143753 | *DEGS1* | -0.37 | 0.3525 |
| ENSG00000063176 | *SPHK2* | 0.25 | 0.4073 |
| ENSG00000139624 | *CERS5* | -0.41 | 0.4584 |
| ENSG00000148154 | *UGCG* | -0.40 | 0.4730 |
| ENSG00000166224 | *SGPL1* | -0.28 | 0.5543 |
| ENSG00000119537 | *KDSR* | 0.15 | 0.5927 |
| ENSG00000100422 | *CERK* | 0.17 | 0.6575 |
| ENSG00000067113 | *PLPP1* | 0.21 | 0.9011 |
| ENSG00000126821 | *SGPP1* | -0.05 | 0.9328 |
| ENSG00000070610 | *GBA2* | 0.02 | 0.9533 |
| ENSG00000162139 | *NEU3* | 0.02 | 0.9688 |
| ENSG00000172292 | *CERS6* | -0.04 | 0.9771 |
| **Gene ID** | **Gene name** | **M** | **p_adj_** |
| ENSG00000090054 | *SPTLC1* | 0.01 | 0.9846 |
| ENSG00000136699 | *SMPD4* | -0.03 | 0.9922 |
| ENSG00000054983 | *GALC* | 0.00 | 0.9950 |

**Figure S7:**


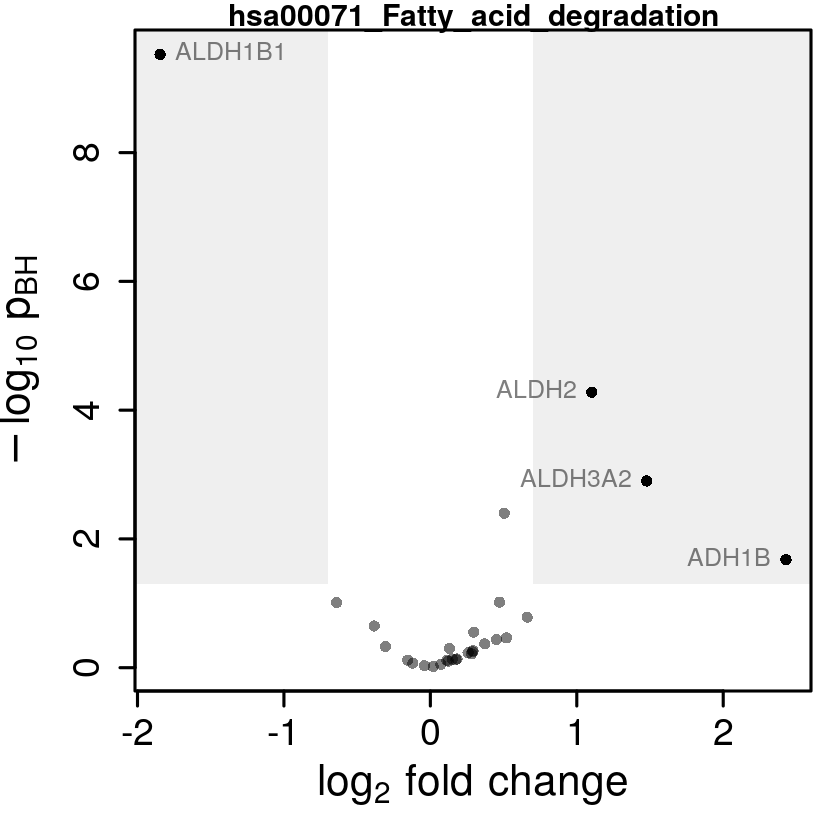


**Figure S7:** Volcano plot representing differential expression of genes involved in the fatty acid degradation pathway. Genes within the grey shaded rectangles are considered significant.

**Table S6:**

| **Gene ID** | **Gene name** | | **M** | **p_adj_** |
| --- | --- | --- | --- | --- |
| ENSG00000137124 | *ALDH1B1* | -1.85 | | 0.0000 |
| ENSG00000111275 | *ALDH2* | 1.10 | | 0.0001 |
| ENSG00000072210 | *ALDH3A2* | 1.48 | | 0.0013 |
| ENSG00000060971 | *ACAA1* | 0.50 | | 0.0040 |
| ENSG00000196616 | *ADH1B* | 2.43 | | 0.0211 |
| ENSG00000198721 | *ECI2* | 0.47 | | 0.0961 |
| ENSG00000087008 | *ACOX3* | -0.64 | | 0.0975 |
| ENSG00000143149 | *ALDH9A1* | 0.66 | | 0.1640 |
| ENSG00000120437 | *ACAT2* | -0.38 | | 0.2251 |
| ENSG00000117054 | *ACADM* | 0.30 | | 0.2809 |
| ENSG00000138029 | *HADHB* | 0.52 | | 0.3442 |
| ENSG00000075239 | *ACAT1* | 0.45 | | 0.3663 |
| ENSG00000151726 | *ACSL1* | 0.37 | | 0.4260 |
| ENSG00000155016 | *CYP2U1* | -0.31 | | 0.4711 |
| ENSG00000127884 | *ECHS1* | 0.13 | | 0.5012 |
| ENSG00000138796 | *HADH* | 0.29 | | 0.5445 |
| ENSG00000196177 | *ACADSB* | 0.29 | | 0.5686 |
| ENSG00000161533 | *ACOX1* | 0.26 | | 0.5883 |
| ENSG00000084754 | *HADHA* | 0.28 | | 0.6096 |
| ENSG00000122971 | *ACADS* | 0.18 | | 0.7349 |
| ENSG00000167315 | *ACAA2* | 0.15 | | 0.7353 |
| ENSG00000068366 | *ACSL4* | 0.17 | | 0.7466 |
| ENSG00000123983 | *ACSL3* | -0.15 | | 0.7632 |
| ENSG00000197894 | *ADH5* | 0.11 | | 0.7677 |
| ENSG00000157184 | *CPT2* | 0.12 | | 0.7935 |
| ENSG00000072778 | *ACADVL* | -0.12 | | 0.8500 |
| ENSG00000110090 | *CPT1A* | 0.07 | | 0.8849 |
| ENSG00000164904 | *ALDH7A1* | -0.04 | | 0.9328 |
| ENSG00000167969 | *ECI1* | 0.02 | | 0.9593 |

**Table S6:** Differential expression of genes involved in Fatty acid degradation. Columns *M* and *p_adj_* contain the log_2_ fold change and adjusted p-values, respectively.

**Figure S8:**


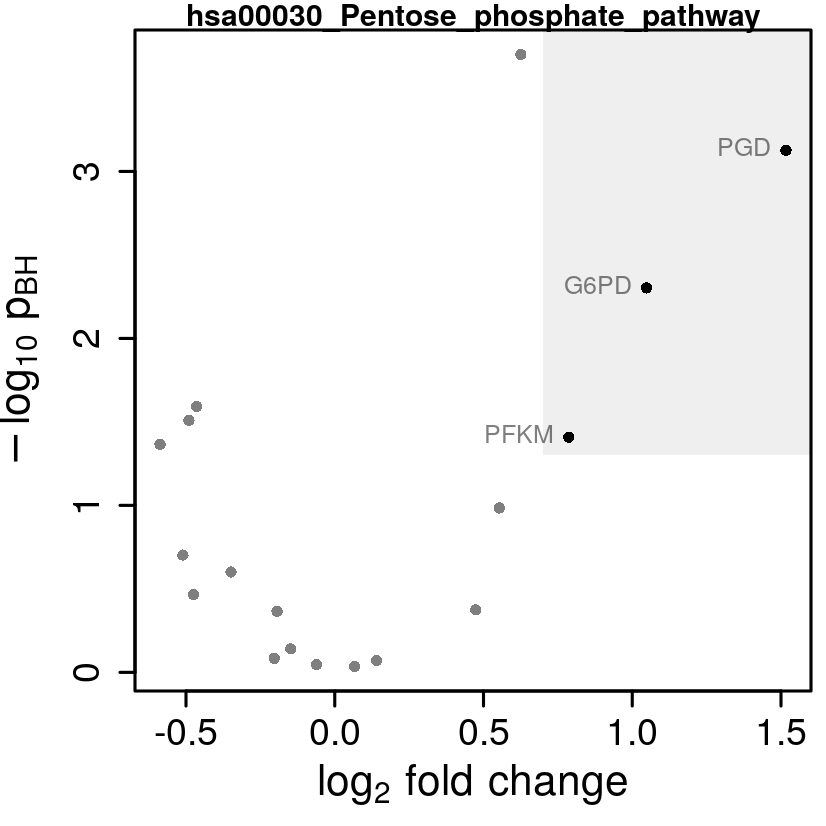


**Figure S8:** Volcano plot representing differential expression of genes involved in the Pentose phosphate pathway. Genes within the grey shaded rectangles are considered significant.

**Table S7:**

| **Gene ID** | **Gene name** | **M** | **p_adj_** |
| --- | --- | --- | --- |
| ENSG00000177156 | *TALDO1* | 0.63 | 0.0002 |
| ENSG00000142657 | *PGD* | 1.52 | 0.0007 |
| ENSG00000160211 | *G6PD* | 1.05 | 0.0050 |
| ENSG00000141959 | *PFKL* | -0.46 | 0.0256 |
| ENSG00000105220 | *GPI* | -0.49 | 0.0310 |
| ENSG00000152556 | *PFKM* | 0.79 | 0.0391 |
| ENSG00000147224 | *PRPS1* | -0.59 | 0.0431 |
| ENSG00000163931 | *TKT* | 0.55 | 0.1036 |
| ENSG00000067057 | *PFKP* | -0.51 | 0.1984 |
| ENSG00000197713 | *RPE* | -0.35 | 0.2507 |
| ENSG00000079739 | *PGM1* | -0.47 | 0.3424 |
| ENSG00000109107 | *ALDOC* | 0.47 | 0.4222 |
| ENSG00000149925 | *ALDOA* | -0.19 | 0.4305 |
| ENSG00000130313 | *PGLS* | -0.15 | 0.7228 |
| ENSG00000023697 | *DERA* | -0.20 | 0.8244 |
| ENSG00000101911 | *PRPS2* | 0.14 | 0.8507 |
| ENSG00000169299 | *PGM2* | -0.06 | 0.8998 |
| ENSG00000049239 | *H6PD* | 0.07 | 0.9225 |

**Table S7:** Differential expression of genes involved in Pentose phosphate pathway. Columns *M* and *p_adj_* contain the log_2_ fold change and adjusted p-values, respectively.

**Figure S9:**


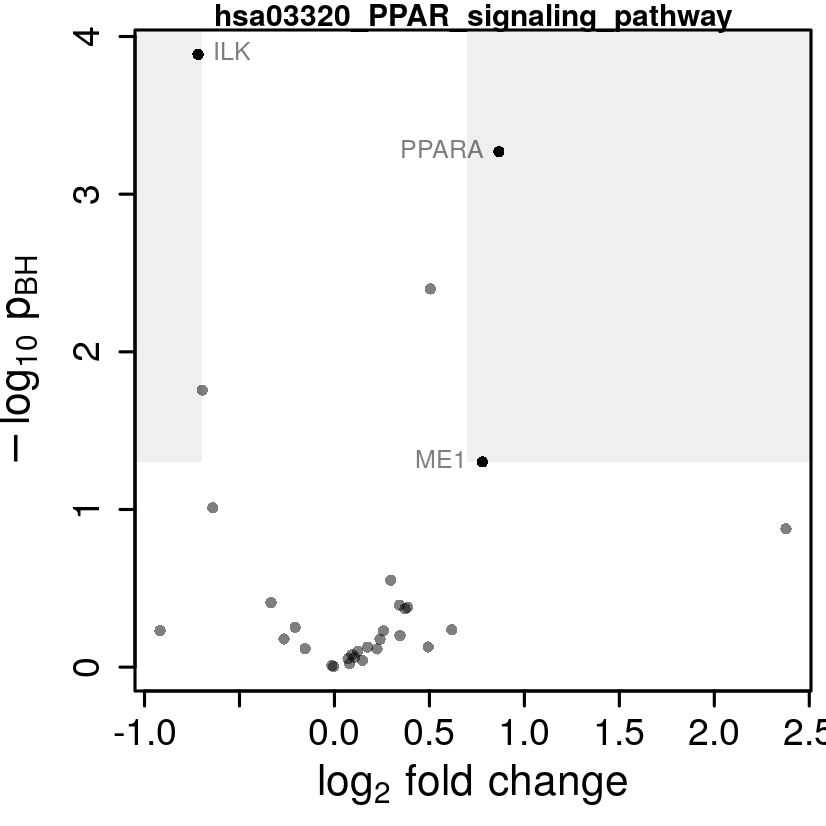


**Figure S9:** Volcano plot representing differential expression of genes involved in the PPAR signaling pathway. Genes within the grey shaded rectangles are considered significant.

**Table S8:**

| **Gene ID** | **Gene name** | **M** | **p_adj_** |
| --- | --- | --- | --- |
| ENSG00000166333 | *ILK* | -0.72 | 0.0001 |
| ENSG00000186951 | *PPARA* | 0.87 | 0.0005 |
| ENSG00000060971 | *ACAA1* | 0.50 | 0.0040 |
| ENSG00000130304 | *SLC27A1* | -0.70 | 0.0175 |
| ENSG00000065833 | *ME1* | 0.78 | 0.0499 |
| ENSG00000087008 | *ACOX3* | -0.64 | 0.0975 |
| ENSG00000196611 | *MMP1* | 2.38 | 0.1327 |
| ENSG00000117054 | *ACADM* | 0.30 | 0.2809 |
| ENSG00000112972 | *HMGCS1* | -0.33 | 0.3892 |
| ENSG00000155368 | *DBI* | 0.34 | 0.4045 |
| ENSG00000186350 | *RXRA* | 0.39 | 0.4173 |
| ENSG00000151726 | *ACSL1* | 0.37 | 0.4260 |
| ENSG00000167114 | *SLC27A4* | -0.21 | 0.5589 |
| ENSG00000099194 | *SCD* | 0.62 | 0.5791 |
| ENSG00000170323 | *FABP4* | -0.92 | 0.5869 |
| ENSG00000161533 | *ACOX1* | 0.26 | 0.5883 |
| ENSG00000145284 | *SCD5* | 0.35 | 0.6305 |
| ENSG00000100889 | *PCK2* | -0.27 | 0.6631 |
| ENSG00000116171 | *SCP2* | 0.24 | 0.6647 |
| ENSG00000135218 | *CD36* | 0.49 | 0.7454 |
| ENSG00000068366 | *ACSL4* | 0.17 | 0.7466 |
| ENSG00000123983 | *ACSL3* | -0.15 | 0.7632 |
| ENSG00000147872 | *PLIN2* | 0.22 | 0.7665 |
| ENSG00000157184 | *CPT2* | 0.12 | 0.7935 |
| ENSG00000204231 | *RXRB* | 0.09 | 0.8330 |
| ENSG00000100979 | *PLTP* | 0.11 | 0.8654 |
| ENSG00000110090 | *CPT1A* | 0.07 | 0.8849 |
| ENSG00000134824 | *FADS2* | 0.15 | 0.9053 |
| ENSG00000167772 | *ANGPTL4* | 0.08 | 0.9495 |
| ENSG00000150991 | *UBC* | -0.01 | 0.9777 |
| ENSG00000140992 | *PDPK1* | 0.00 | 0.9910 |
|  |  |  |  |
|  |  |  |  |

**Table S8:** Differential expression of genes involved in PPAR signaling pathway. Columns *M* and *p_adj_* contain the log_2_ fold change and adjusted p-values, respectively.

**Figure S10:**


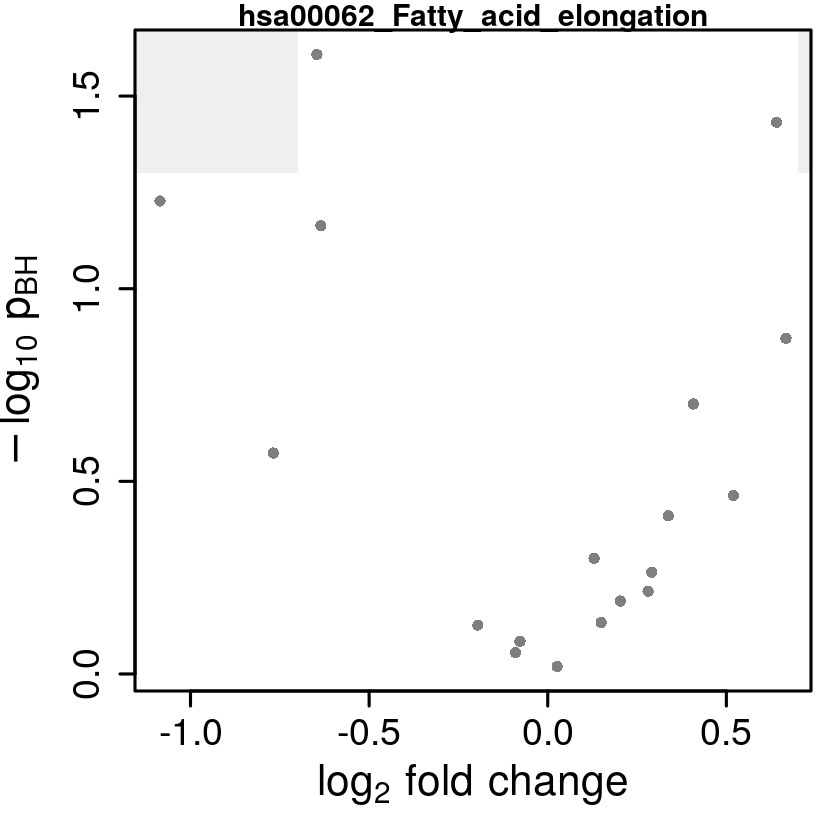


**Figure S10:** Volcano plot representing differential expression of genes involved in the Fatty acid elongation pathway. Genes within the grey shaded rectangles are considered significant.

**Table S9:**

| **Gene ID** | **Gene name** | **M** | **p_adj_** |
| --- | --- | --- | --- |
| ENSG00000119673 | *ACOT2* | -0.65 | 0.0247 |
| ENSG00000131238 | *PPT1* | 0.64 | 0.0370 |
| ENSG00000165996 | *HACD1* | -1.09 | 0.0592 |
| ENSG00000206527 | *HACD2* | -0.64 | 0.0686 |
| ENSG00000119915 | *ELOVL3* | 0.67 | 0.1345 |
| ENSG00000221988 | *PPT2* | 0.41 | 0.1991 |
| ENSG00000097021 | *ACOT7* | -0.77 | 0.2670 |
| ENSG00000138029 | *HADHB* | 0.52 | 0.3442 |
| ENSG00000170522 | *ELOVL6* | 0.34 | 0.3885 |
| ENSG00000127884 | *ECHS1* | 0.13 | 0.5012 |
| ENSG00000138796 | *HADH* | 0.29 | 0.5445 |
| ENSG00000084754 | *HADHA* | 0.28 | 0.6096 |
| ENSG00000159445 | *THEM4* | 0.20 | 0.6466 |
| ENSG00000167315 | *ACAA2* | 0.15 | 0.7353 |
| ENSG00000012660 | *ELOVL5* | -0.20 | 0.7468 |
| ENSG00000074696 | *HACD3* | -0.08 | 0.8224 |
| ENSG00000149084 | *HSD17B12* | -0.09 | 0.8804 |
| ENSG00000116353 | *MECR* | 0.03 | 0.9564 |

**Table S9:** Differential expression of genes involved in Fatty acid elongation. Columns *M* and *p_adj_* contain the log_2_ fold change and adjusted p-values, respectively.

**Figure S11:**


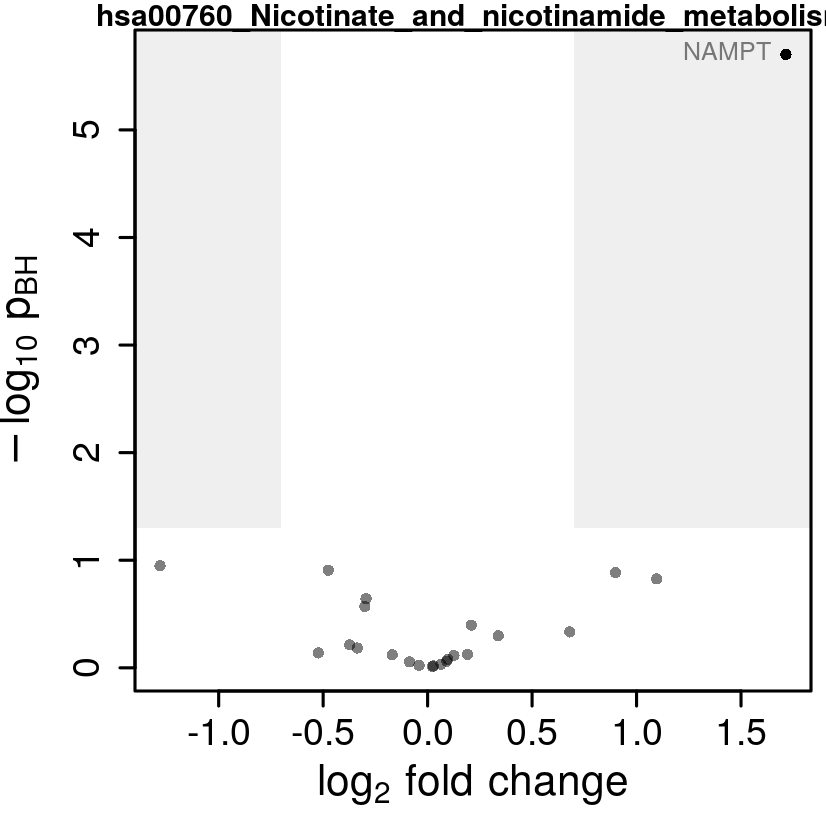


**Figure S11:** Volcano plot representing differential expression of genes involved in the Nicotinate and nicotinamide metabolism pathway. Genes within the grey shaded rectangles are considered significant.

**Table S10:**

| **Gene ID** | **Gene name** | **M** | **p_adj_** |
| --- | --- | --- | --- |
| ENSG00000105835 | *NAMPT* | 1.72 | 0.0000 |
| ENSG00000109743 | *BST1* | -1.28 | 0.1127 |
| ENSG00000125458 | *NT5C* | -0.47 | 0.1238 |
| ENSG00000135318 | *NT5E* | 0.90 | 0.1306 |
| ENSG00000138356 | *AOX1* | 1.10 | 0.1490 |
| ENSG00000077463 | *SIRT6* | -0.29 | 0.2271 |
| ENSG00000152620 | *NADK2* | -0.30 | 0.2695 |
| ENSG00000068903 | *SIRT2* | 0.21 | 0.4013 |
| ENSG00000197594 | *ENPP1* | 0.68 | 0.4639 |
| ENSG00000106733 | *NMRK1* | 0.34 | 0.5037 |
| ENSG00000166741 | *NNMT* | -0.37 | 0.6123 |
| ENSG00000147813 | *NAPRT* | -0.34 | 0.6544 |
| ENSG00000103485 | *QPRT* | -0.52 | 0.7270 |
| ENSG00000187531 | *SIRT7* | 0.19 | 0.7488 |
| ENSG00000096717 | *SIRT1* | -0.17 | 0.7559 |
| ENSG00000124523 | *SIRT5* | 0.13 | 0.7677 |
| ENSG00000198805 | *PNP* | 0.10 | 0.8318 |
| ENSG00000173614 | *NMNAT1* | 0.09 | 0.8697 |
| ENSG00000008130 | *NADK* | -0.09 | 0.8802 |
| ENSG00000076685 | *NT5C2* | 0.06 | 0.9246 |
| ENSG00000112992 | *NNT* | -0.04 | 0.9465 |
| ENSG00000142082 | *SIRT3* | 0.03 | 0.9597 |
| ENSG00000172890 | *NADSYN1* | 0.02 | 0.9726 |

**Table S10:** Differential expression of genes involved in Nicotinate and nicotinamide metabolism. Columns *M* and *p_adj_* contain the log_2_ fold change and adjusted p-values, respectively.

**Figure S12:**

**Figure S12:** Effect of MitoTempo on mitochondrial ROS and intracellular lipid accumulation evaluated in ACM CStCs after 7 days ADIPO treatment. **(A)** Representative immunofluorescence images of mitochondrial ROS stained with MitoSOX™ in ACM CStCs in absence or presence of MitoTempo. Original Magnification 40X; scale bar 100 µm. On the right, scatter plot reporting the quantification of MitoSOX™ integrated intensity normalized on the total number of nuclei per field of ACM CStCs in the two experimental conditions. N=4 independent ACM patients, with 7-9 observations for each treatment group. Random intercept model p= 3.1x10^-7^ vs ADIPO. **(B)** Representative immunofluorescence images of intracellular lipid droplets stained with BODIPY 493/503 in ACM CStCs in absence or presence of MitoTempo. Original Magnification 40X; scale bar 100 µm. On the right, scatter plot showing the quantification of BODIPY493/503 integrated intensity normalized on the total number of nuclei per field of ACM CStCs in the two experimental conditions. N=4 independent ACM patients, with 7-15 observations available for each treatment group. Random intercept model p= 4.4x10^-6^ vs ADIPO.

**Figure S13:**

**Figure S13:** Mitochondrial ultrastructure and networks analyses. (A) Representative images of mitochondrial ultrastructure analyzed by TEM microscopy in CTR and ACM CStCs after 7 days in ADIPO medium. m: mitochondria (B) Representative images of mitochondrial network analyzed in CTR and ACM CStCs after 7 days in ADIPO medium. Original Magnification 63X. On the right, scatter plot reporting the ratio between mitochondrial network length and unconnected part. Results are based on N=4 CTR independent individuals and N=3 ACM independent patients with 2-3 microscopy fields available for each treatment group. Random intercept model, ns, p= 0.719 vs ACM.
